# Excited-State Identification of a Nickel-Bipyridine Photocatalyst by Time-Resolved X-ray Absorption Spectroscopy

**DOI:** 10.1021/acs.jpclett.4c00226

**Published:** 2024-05-01

**Authors:** Rachel F. Wallick, Sagnik Chakrabarti, John H. Burke, Richard Gnewkow, Ju Byeong Chae, Thomas C. Rossi, Ioanna Mantouvalou, Birgit Kanngießer, Mattis Fondell, Sebastian Eckert, Conner Dykstra, Laura E. Smith, Josh Vura-Weis, Liviu M. Mirica, Renske M. van der Veen

**Affiliations:** † Department of Chemistry, University of Illinois at Urbana—Champaign, Urbana, Illinois 61801, United States; ‡ Department of Atomic-Scale Dynamics in Light-Energy Conversion, Helmholtz-Zentrum Berlin für Materialien und Energie, Berlin 14109, Germany; § Institute of Optics and Atomic Physics, Technische Universität Berlin, Berlin 10623, Germany

## Abstract

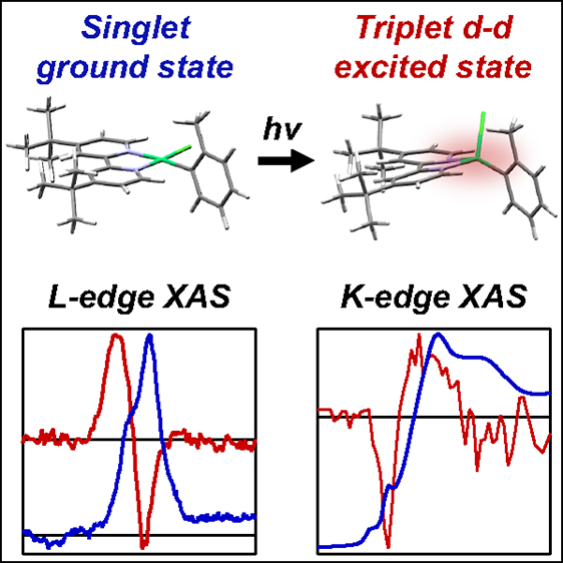

Photoassisted catalysis using Ni complexes is an emerging field for cross-coupling reactions in organic synthesis. However, the mechanism by which light enables and enhances the reactivity of these complexes often remains elusive. Although optical techniques have been widely used to study the ground and excited states of photocatalysts, they lack the specificity to interrogate the electronic and structural changes at specific atoms. Herein, we report metal-specific studies using transient Ni L- and K-edge X-ray absorption spectroscopy of a prototypical Ni photocatalyst, (dtbbpy)Ni(*o*-tol)Cl (dtb = 4,4′-di-*tert*-butyl, bpy = bipyridine, *o*-tol = *ortho*-tolyl), in solution. We unambiguously confirm via direct experimental evidence that the long-lived (∼5 ns) excited state is a tetrahedral metal-centered triplet state. These results demonstrate the power of ultrafast X-ray spectroscopies to unambiguously elucidate the nature of excited states in important transition-metal-based photocatalytic systems.

Over the past several years, light-driven bond-forming reactions have become ubiquitous in synthetic organic chemistry.^1,2^ Of particular interest is the merger of photochemistry with transition metal (TM) cross-coupling catalysis (e.g., photoredox catalysis), wherein a precious-metal photosensitizer absorbs light and undergoes electron transfer with a TM catalyst, forming a highly reactive high-valent ground-state TM species.^3−5^ A more attractive way to activate molecular catalysts, however, is direct photoexcitation of the metal center and its tunable ligand system. This strategy eliminates the need for an exogenous, precious-metal photosensitizer to absorb light, unlocks potentially new reactivity of the photoexcited metal complex that is not accessible by ground-state photoredox mechanisms, and enables more control of excited-state reactivity by tuning the ligand system.^6^ In particular, Ni complexes display unique photoreactivity compared to second- and third-row transition metal species, with the ability to adopt oxidation states between 0 and IV and undergo both one- and two-electron chemistry^7−13^

The unambiguous determination of the nature and lifetime of excited states in photocatalysis is crucial, as these states may be indirectly or directly responsible for the reaction outcome (i.e., product yield, selectivity, specificity, and byproducts). Long-lived metal-to-ligand charge transfer (MLCT) states, for example, are well-known for undergoing photoredox reactions given the charge-transfer nature of the excited state.^14^ Ligand-field (LF) excited states, on the other hand, typically display bond dissociation and cleavage reactivity leading to photosubstitution reactions and photorelease of small molecules.^7,15^ Targeted modifications of the ligand system of TM complexes can be used to tune the energy landscape and properties of the various excited states.^16−18^ A first prerequisite step in this endeavor is the unambiguous identification of the excited states of relevant photocatalytic systems.

In contrast to their second- and third-row transition metal counterparts, the ultrafast photophysics of Ni photocatalysts has only been scarcely studied.^15,19,20^ In this study, we aim to elucidate the nature of the long-lived excited state of a characteristic Ni photocatalyst, (dtbbpy)Ni(*o*-tol)Cl (dtb = 4,4′-di-*tert*-butyl, bpy = bipyridine, *o*-tol = *ortho*-tolyl), upon light excitation. This molecule represents the proposed ground-state analog to the intermediate species formed in the Ni photocatalytic cycle for carbon–heteroatom bond formation reactions and has been shown to perform C–O cross-coupling reactions via direct light excitation.^19,20^ Previously, Doyle and co-workers reported that this complex exhibits an excited state that decays back to the ground state on the time scale of 3–8 ns, depending on the solvent.^19,20^ They initially assigned this state to an MLCT state based on density function theory (DFT) calculations.^19^ In a later study, they provide compelling evidence for the decay of the MLCT state within ∼10 ps via time-resolved infrared (IR) spectroscopy, and they suggested that the long-lived excited state is instead a tetrahedral LF ^3^dd state that was predicted by DFT.^20^ The experimental evidence for the ^3^dd state was only indirect since a distant IR reporter substituent group on the bpy ligand was probed rather than bonds directly connected to the Ni center. In this work, we sought to provide this direct piece of experimental evidence by employing a spin-, charge-, and symmetry-sensitive spectroscopy tool, namely, ultrafast X-ray absorption spectroscopy (XAS), that directly probes the metal atom.^21−24^ By using picosecond-resolved Ni L- and K-edge X-ray transient absorption (XTA) spectroscopy, we unambiguously determine the identity of the ∼5 ns excited state of (dtbbpy)Ni(*o*-tol)Cl as a tetrahedral ^3^dd state. While ultrafast K-edge spectroscopy is well-established for Ni coordination compounds,^25−28^ the Ni L-edge in the soft X-ray range has been, to our knowledge, unexplored so far in this time regime. Ultrafast soft XAS in general is still an underutilized tool in photocatalysis, partly due to the rather complex experimental implementation.^21,29^ Our results demonstrate the potential of ultrafast core-level spectroscopies to identify the nature of excited states in photocatalysis, paving the way toward experiments on more complex TM-based photocatalytic materials and under realistic catalytic environments.

## L-Edge X-ray Absorption Spectroscopy

In first-row TM complexes, soft X-ray L_2,3_-edge spectroscopy locally probes the empty 3d density of states at the metal center through dipole-allowed 2p → 3d transitions and is thereby sensitive to metal–ligand covalency, the metal oxidation state, spin state, and local coordination symmetry.^30−33^ We performed solution-phase Ni L-edge spectroscopy in transmission mode to characterize the ground state of (dtbbpy)Ni(*o*-tol)Cl in dimethylformamide (DMF) (see Supporting Information (SI) for the synthetic details). The Ni L_2,3_-edge transitions lie at approximately 850 eV (2p_3/2_-core excitation, L_3_ edge) and 870 eV (2p_1/2_-core excitation, L_2_ edge). Because soft X-rays at these energies are heavily absorbed and scattered by matter, a very thin liquid sheet of sample must be formed in a vacuum chamber. For this purpose, we used a vacuum-compatible colliding liquid jet with a sheet thickness of approximately 2 μm as part of the *nmTransmissionNEXAFS* end station at beamline UE52_SGM of the BESSY II synchrotron facility^34,35^ (see SI section S2 for more details).

**Figure 1:**
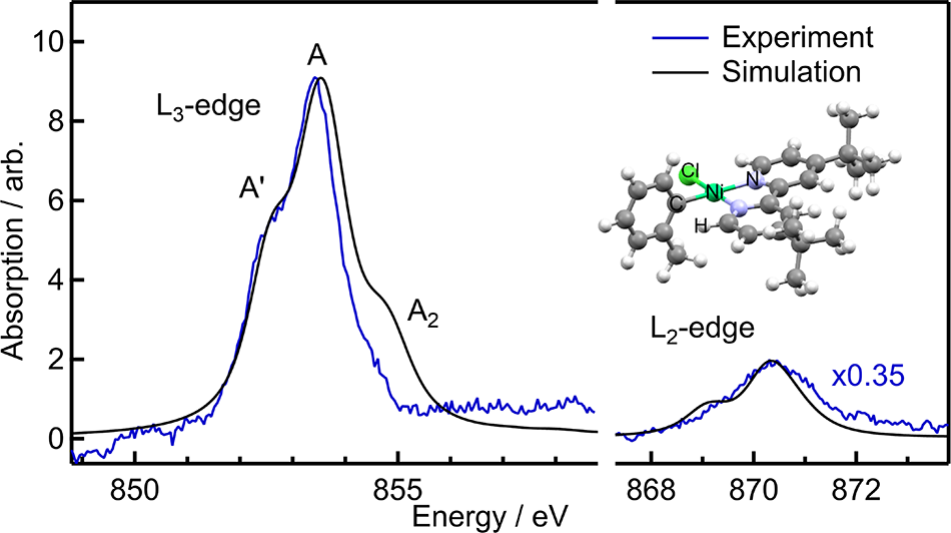
Experimental L_3_- and L_2_-edge XAS spectra (blue trace) with the simulated spectrum using the CTM4XAS code in black. The simulated spectrum is arbitrarily scaled to match the intensity of the L_3_-edge white line, and the L_2_-edge experimental data are arbitrarily scaled to match the intensity of the simulated L_2_-edge peak using the same parameters.

Figure 1 shows the ground-state L_2,3_-edge spectra of 20 mM (dtbbpy)Ni(*o*-tol)Cl in DMF, together with the simulated spectrum. The L_3_-edge spectrum contains one main peak (A) at 853.4 eV, with a prominent shoulder (A′) on the red side at approximately 852.6 eV and a weaker shoulder (A_2_) on the blue side at approximately 854.6 eV. The L_2_-edge spectrum features one main peak; the lower energy shoulder visible in the simulation could not be clearly resolved.

Simulations were performed using the semiempirical ligand-field multiplet software package CTM4XAS^36,37^ (see SI section S2.1 for details). This code uses a parametric Hamiltonian that represents the ligand field strength and symmetry, describing the ground state as a linear combination of ligand-field configurations such as Ψ = α_1_d_π_^4^d_*xy*_^2^d_*z*^2^_^2^d_*x*^2^–*y*^2^_^0^ + α_2_d_π_^4^d_*xy*_^2^d_*z*^2^_^1^d_*x*^2^–*y*^2^_^1^ + ..., where the energy of each configuration is given by the ligand field parameters 10Dq, Ds, and Dt for a system with D_4h_ symmetry. In systems with appreciable covalency, additional configurations accounting for charge transfer to and from a ligand are added and parametrized by the energy difference and coupling strength between the d^N^L and d^N+1^L^+^ states. These configurations are mixed via spin–orbit coupling, resulting in a manifold of electronic states. Transition dipole matrix elements are then calculated between the 3d^N^ ground state and 2p^5^3d^N+1^ excited states to produce an XAS spectrum.

Beginning with ligand-field parameters developed for low-spin Ni(II)octaethylporphyrin and including charge transfer parameters to account for covalency,^38,39^ we adjusted the ligand field parameters until we achieved a close match between the simulation and the experimental spectrum. The best match between simulation and experiment is shown in Figure 1 (for more details on the parameters used, see SI section S2.1). The energy spacing between the main peak A and the low-energy shoulder A′ as well as their intensity ratio show an excellent match to the experiment, though the strength of the high-energy shoulder A_2_ is overestimated in the simulation of the L_3_-edge spectrum.

We note that the L_2,3_ spectrum of (dtbbpy)Ni(*o*-tol)Cl in powder form is nearly identical to the solution-phase spectrum (Figure S1). Since the crystal structure shows a nearly square-planar geometry around the Ni center,^19^ this rules out the possibility of solvent coordination and significant distortions away from square-planar symmetry in solution.

## Transient L-edge XAS

We performed laser pump–soft X-ray probe experiments at the UE52_SGM beamline at BESSY II^34^ in a 20 mM solution of (dtbbpy)Ni(*o*-tol)Cl in DMF and laser excitation at 343 nm delay (350 fs pump, 208 kHz, 3 mJ/cm^2^ absorbed fluence; see SI section S2 for more experimental details). Previous transient optical and IR absorption experiments were done with excitation of the complex at >400 nm and showed features of vibrational cooling in the MLCT manifold on the ∼1 ps time scale, decay into an intermediate excited state on the 5–10 ps time scale, and back-relaxation to the ground state on the few nanosecond time scale.^20^ In the L-edge XTA experiments reported herein, the pump wavelength was chosen to be 343 nm, overlapping with the red edge of the ligand π → π* transition (Figure S8). Our optical transient absorption (OTA) data (SI Section S4.1) show that for this excitation wavelength, the MLCT states are populated within the instrumental response (120 fs), and the consequent dynamics are similar to those for longer excitation wavelengths. We conclude that in our XTA experiments at a ∼100 ps time delay, the same long-lived state with a lifetime of ∼5 ns is populated as in previous studies with lower-energy excitation.^19,20^

**Figure 2:**
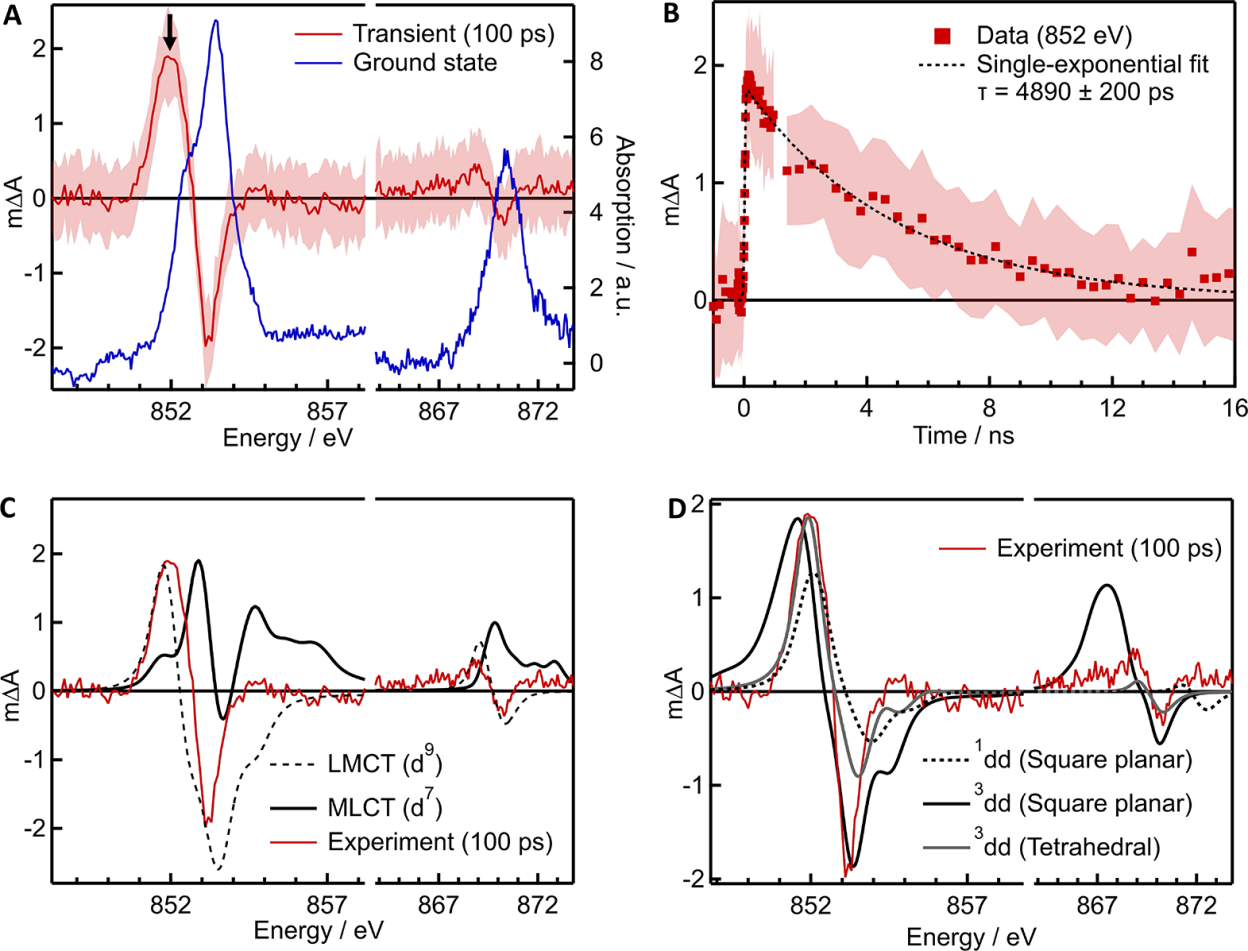
(A) L_2,3_-edge transient spectra of 20 mM (dtbbpy)Ni(*o*-Tol)Cl in DMF at ∼100 ps after photoexcitiation at 343 nm (red) together with the ground-state spectra in blue. (B) Kinetic trace collected at 852 eV (peak of the L_3_ transient, black arrow in panel A) with the single-exponential fit shows in the dashed line. (C) Simulated difference spectra using d^7^ (solid black, “MLCT state”) and d^9^ (dashed black, “LMCT state”) electron configurations for Ni, together with the experimental transient spectra (red; same as in A). (D) Simulated difference spectra using high-lying ^3^dd (solid black) and ^1^dd (dashed black) states with the square-planar ground-state ligand field symmetry and parameters, together with the experimental transient spectra (red; same as in A). The simulated difference spectrum using a tetrahedral ligand field is shown in gray. Shaded areas in A and B denote ± standard deviation. The simulated spectra in C and D have been arbitrarily scaled to match the intensity of the experimental data at 853.4 eV.

The transient (laser on minus laser off) Ni L_2,3_-edge spectra at ∼100 ps are shown in Figure 2A, together with the ground-state spectra for comparison. On first sight, both edges seem to shift to lower energy upon photoexcitation, which may be due to changes in charge density at the Ni center^40,41^ or changes in ligand-field strength and/or symmetry.^42,43^ Kinetic monitoring at 852 eV, corresponding to the peak of the L_3_-edge transient, yields a single-exponential decay with a decay time of 4890 ± 200 ps (Figure 2B), in excellent agreement with the ground-state recovery time determined in OTA experiments (Figures S10 and S11). This confirms that we are probing the longest-lived excited state of (dtbbpy)Ni(*o*-tol)Cl whose nature we seek to unambiguously determine here.

To this extent, we investigated several excited-state scenarios using the ligand-field multiplet method described above (details are provided in the SI section S2.1). First, we calculated the spectra for Ni with d^7^ and d^9^ electron configurations, instead of d^8^ for Ni(II), in order to qualitatively simulate square-planar excited states with MLCT or LMCT character (Figure 2C). All simulations were performed with the same ligand-field and charge-transfer parameters as the best-match simulation for the ground state (Figure 1); however, the d^9^ state did not include charge transfer, as the addition of charge transfer would invoke a d^10^ state, which has no L_2,3_-edge intensity. The simulated ground-state spectrum was subtracted to generate difference spectra to compare to the experimental transient spectrum at ∼100 ps after photoexcitation. Overall, the spectra of states with different d-electron counts do not do a poor job of simulating the experimental data. Although the d^9^ (“LMCT”) state features a red shift, the simulated spectrum is much broader than the experimental spectrum.

Next, d^8^ square-planar excited states were considered (dd states; Figure 2D). The simulated difference spectrum for a square-planar excited ^1^dd state features a red shift, as seen in the experiment, but it is significantly smaller than the observed experimental red shift. On the other hand, the simulated difference spectrum for a square-planar excited ^3^dd state exhibits a reasonable match at the L_3_ edge, but the width of the transient features and the red shift at the L_2_-edge are heavily overestimated. Finally, we considered a geometric change in the excited state. DFT calculations indicate that the lowest-lying triplet state has tetrahedral geometry,^20^ and so we simulated a triplet state with a tetrahedral ligand field without charge transfer character (Figure 2D). This excited-state simulation exhibits the best match to the experiment at both edges in terms of the peak position and width of the transient features. The discrepancy in bleach intensity at the L_3_-edge between the experiment and the simulation may be due to the assumption that the CTM4XAS program requires the selection of a specific geometry and cannot account for the true C_1_ symmetry of the complex, nor for any geometries deviating from pure point-group symmetries (e.g., slight distortions away from square-planar geometry as suggested by the crystal structure of (dtbbpy)Ni(*o*-tol)Cl)^19^). Our L-edge XTA data thus strongly support the assignment of the long-lived excited state of (dtbbpy)Ni(*o*-tol)Cl as the tetrahedral metal-centered ^3^dd state predicted by DFT (SI section S6), in agreement with the proposed state assignment by Doyle et al.^20^

## Transient K-Edge XAS

**Figure 3:**
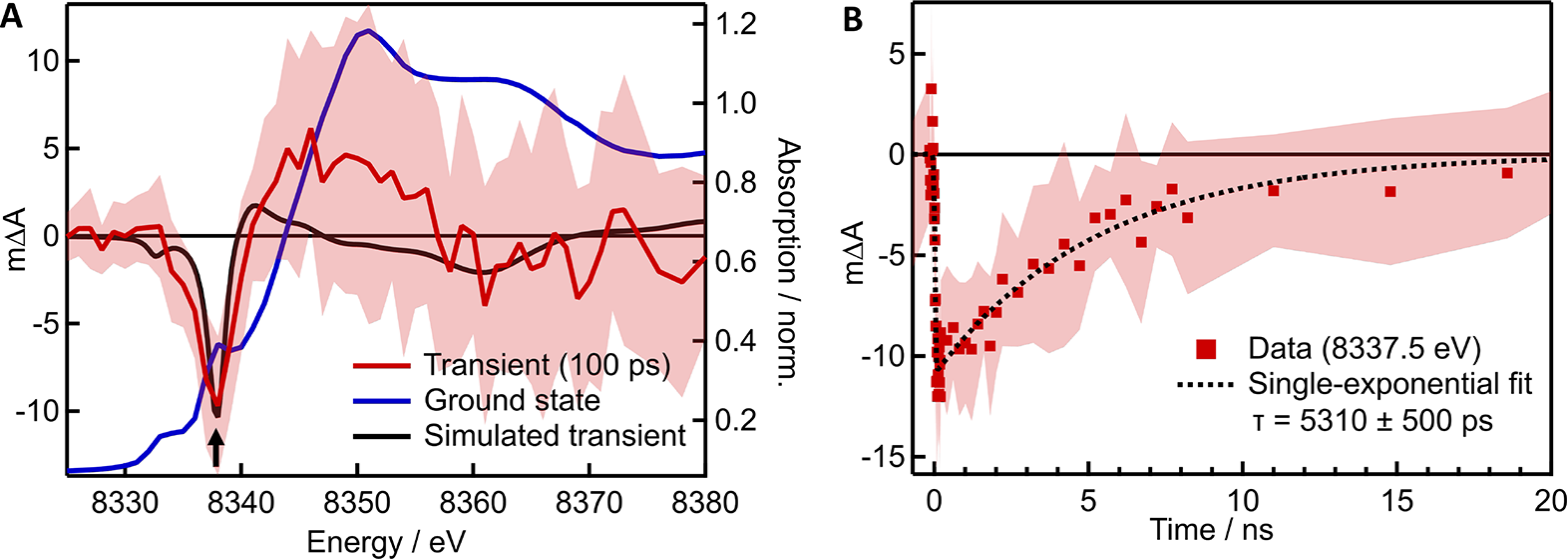
(A) Ground-state K-edge spectrum in blue with the transient X-ray spectrum measured at a 100 ps delay after photoexcitiation at 515 nm in red, and the simulated transient spectrum in black. The latter has been scaled by a factor of 0.01 to match the amplitude of the bleach feature at 8337.5 eV. (B) Kinetic monitoring at 8337.5 eV (black arrow in panel A) showing a decay time of 5310 ± 500 ps, in good agreement with optical and L-edge measurements. Shaded areas denote ± 1 standard deviation.

We performed static and transient XAS measurements of the (dtbbpy)Ni(*o*-tol)Cl complex (5 mM in DMF) at the Ni K-edge at beamline 11-ID-D of the Advanced Photon Source (APS;^44^ see SI section S5 for experimental details). K-edge spectra of first-row TM complexes involve the excitation of a 1s electron to empty 4p states and are highly sensitive to local geometry about the metal center.^45^ The K-edge data are shown in Figure 3. The static spectrum (Figure 3A) features a pre-edge peak corresponding to the 1s → 3d transition at 8333 eV^26^ and a shoulder at the low-energy side of the white line at 8337.5 eV, which is assigned to the 1s → 4p_*z*_ transition.^28,46^ The Ni 4p_*x*,*y*_ orbitals are higher in energy than the 4p_*z*_ orbitals in square-planar complexes due to strong σ interactions between the 4p_*x*,*y*_ orbitals and the ligand 2s orbitals.^27^

The transient K-edge spectrum recorded at ∼100 ps after photoexcitation (515 nm, 20 mJ/cm^2^ absorbed fluence, 120 fs) is shown in Figure 3A. The transient spectrum exhibits bleaching of the 1s → 4p_*z*_ rising-edge feature and an increase in intensity at the white line (1s → 4p_*x*,*y*_; Figure 3A). This suggests that in the excited state the 1s → 4p_*z*_ absorption band shifts to higher energies due to destabilization of the 4p_*z*_ orbital. The latter is consistent with the geometric reorganization to a tetrahedral ligand field in the ^3^dd excited state, in which all Ni 4p orbitals are hybridized with 3d orbitals and are degenerate. The kinetics were monitored at 8337.5 eV corresponding to the peak of the 4p_*z*_ bleach (Figure 3B). A single-exponential decay with a time constant of 5310 ± 500 ps is obtained, in good agreement with both the time-resolved optical (section S4.1) and L-edge (Figure 2B) data.

To corroborate this interpretation, we performed simulations of the Ni K-edge spectrum using the real-space, full-potential FDMNES code.^47,48^ The simulated difference spectrum is generated by subtracting the normalized ground-state simulation from the normalized excited-state simulation using the geometry-optimized square-planar and tetrahedral geometries from DFT as input structures, respectively (SI section S6). The resulting (scaled) difference spectrum shows a reasonable match to the experimental transient spectrum with a decrease in intensity at the 1s → 4p_*z*_ peak and a slight increase in intensity in the white line region (Figure 3A). The simulated transient is scaled by a factor of 0.01 to match the intensity of the ground-state bleach, which indicates an excitation fraction of ∼1%. However, this factor is artificially low due to the overestimated strength of the 1s → 4p_*z*_ transition in the ground-state simulation by approximately a factor of 10 (Figure S13). The excitation fraction is therefore actually closer to ∼10%.

Qualitatively, these observations are similar to the transient K-edge spectra observed for Ni(II) porphyrins or Ni(II) phthalocyanines in coordinating solvents.^25,26^ The long-lived transient states in coordinating solvents of Ni(II) porphyrins and phthalocyanines involve solvent coordination to the Ni(II) center to form an octahedral complex from a square-planar ground state, destabilizing the 4p_*z*_ orbital as evidenced by the large blue-shifting of the 1s → 4p_*z*_ orbital in the excited (ligated) state.

Using a combination of transient picosecond-resolved L-edge and K-edge X-ray spectroscopy, we provide direct evidence that the lowest excited state of (dtbbpy)Ni(*o*-tol)Cl is a tetrahedral triplet ^3^dd state with a lifetime of ∼5 ns for back-relaxation to the ground state. To our knowledge, this is the first report of a time-resolved Ni L-edge experiment on a photocatalytic complex at a relatively low concentration in solution. This has implications not only for future experiments on first-row transition metal photocatalysts but also for the ability to access ligand K-edges that lie in the soft X-ray region.^49−51^ Time-resolved nitrogen K-edge measurements, for example, can provide previously unexplored insight into bonding in the excited-state, bond cleavage reactions, and other metal–ligand interactions that may be relevant for photocatalysis.^52−54^

The long-lived metal-centered state could indicate that catalysis with first-row transition metal systems is performed via unique mechanisms compared with second- and third-row counterparts. Recent results suggest that metal-centered states of first-row transition metal complexes may be able to undergo bimolecular oxidative electron transfer,^55^ which is a distinct avenue of reactivity. Building on the success of performing time-resolved soft X-ray spectroscopy in relatively dilute solution, further exploration of this mechanism could involve direct tracking of the substrate molecules as they undergo electron or energy transfer from the excited state of the photocatalyst.

## Supporting Information Available

Synthetic details, UV/visible absorption spectroscopy and TDDFT spectra, optical transient absorption spectroscopy and related fits, L-edge experimental details, K-edge experimental details, ^1^H NMR spectra (PDF)
