# Supplementary material for: Excited-State Identification of a Nickel-Bipyridine Photocatalyst by Time-Resolved X-ray Absorption Spectroscopy
Source: J Phys Chem Lett. 2024 May 1;15(18):4976–82. doi: 10.1021/acs.jpclett.4c00226 (PMC11089568; doi:10.1021/acs.jpclett.4c00226)
Supplement: Supplementary file 1 — jz4c00226_si_001.pdf [file jz4c00226_si_001.pdf]

Supplementary Information

for

Excited-State Characterization of a Nickel-Bipyridine Photocatalyst by Time-Resolved X-ray Absorption Spectroscopy

Rachel F. Wallick[1], Sagnik Chakrabarti[1], John H. Burke[1], Richard Gnewkow[2,3], Ju Byeong Chae[1], Thomas C. Rossi[2], Ioanna Mantouvalou[2,3], Birgit Kanngießer[2,3], Mattis Fondell[2], Sebastian Eckert[2], Conner Dykstra[1], Laura E. Smith[1], Josh Vura-Weis[1]*, Liviu M. Mirica[1]*, Renske M. van der Veen[1,2,3]*

[1]Department of Chemistry, University of Illinois at Urbana-Champaign, Urbana, Illinois, 61801, USA

[2]Helmholtz-Zentrum Berlin für Materialien und Energie, Berlin, 12489, Germany

[3]Institute of Optics and Atomic Physics, Technische Universität Berlin, Berlin, 10623, Germany

*vuraweis@illinois.edu; mirica@illinois.edu; renske.vanderveen@helmholtz-berlin.de

Contents

S1. Synthesis of (dtbbpy)Ni(*o*-tol)Cl.....2

S2. L-edge absorption spectroscopy.....3

S2.1 CTM Simulations.....4

S3. NMR Characterization.....6

S4. Optical absorption spectroscopy.....11

S4.1 OTA in DMF.....13

S5. K-edge X-ray absorption spectroscopy.....16

S6. Density Functional Theory Calculations.....17

S7. References.....25

S1. Synthesis of (dtbbpy)Ni(*o*-tol)Cl

All reagents were commercially available from Aldrich or Cambridge Isotope Laboratories and were used as received without further purification. All synthesis was performed in a dry N_2 environment. Deuterated solvents were dried over 2 Å molecular sieves for 48 hours, except for the "dry" and "wet" measurements, in which the solvents were dried for 10 days over sieves or were used without further purification, respectively.

Published methods for the synthesis of (dtbbpy)Ni(*o*-tol)Cl were followed[1]. Briefly, in a nitrogen-filled glove box, a 100 mL round bottom flask equipped with a stir bar was charged with $Ni(COD)_2$ (5.5 g, 20 mmol), 4,4'-di-tert-butyl-2,2'-bipyridine (dtbbpy, 5.4 g, 20 mmol) and tetrahydrofuran (THF) (30 mL). The resulting dark purple solution was stirred for 1 hour at room temperature. 2-chlorotoluene (30 mL) was added to the reaction flask and left to stir overnight. Hexanes were added to the resulting dark red solution and the reaction flask was kept in a -35 ºC freezer for 30 minutes. The precipitate was collected and washed with hexanes and ether. The residual solvent was removed under reduced pressure to obtain the desired product with a yield of 86%. The obtained product has identical spectroscopic properties to those reported previously.

To ascertain the purity of the compound synthesized, elemental analysis was performed, the results of which are summarized in Table S1 below:

	C	H	N	Cl
Theoretical 100% (dtbbpy)Ni(*o*-tol)Cl	66.19%	6.89%	6.17%	7.81%
Measured	65.96%	6.91%	6.20%	8.86%
Difference	-0.23%	0.02%	0.03%	1.05%

Table S1. Combustion analysis results of (dtbbpy)Ni(*o*-tol)Cl for C, H, N, and Cl. The CHN results are all within the 0.4% tolerance interval within the standard deviation of two runs on the same sample. The Cl shows a ~1% difference, which is examined below.

Because of the higher measured halide percentage, we investigated the possibility of a mixture of (dtbbpy)Ni(*o*-tol)Cl and (dtbbpy)$NiCl_2$. (dtbbpy)$NiCl_2$ is expected to form in small amounts from the synthesis of the complex. We computed the theoretical values for different ratios of (dtbbpy)Ni(*o*-tol)Cl/(dtbbpy)$NiCl_2$ and compared them to the measured values (Table S2). Generally, the addition of (dtbbpy)$NiCl_2$ increases the elemental percentage of Cl. For 5-10% of (dtbbpy)$NiCl_2$ the Cl percentages better matches the measured percentage. We note that halide combustion analysis is generally more difficult to obtain than CHN analysis and the deviations may be due to random error in the experiment, which has been reported to happen in over 10% of all combustion analysis studies[2].

	C	H	N	Cl
Measured	65.96%	6.91%	6.20%	8.86%
Theo. 70/30% (dtbbpy)Ni(*o*-tol)Cl/(dtbbpy)$NiCl_2$	62.94%	6.94%	6.41%	10.55%
Theo. 90/10% (dtbbpy)Ni(*o*-tol)Cl/(dtbbpy)$NiCl_2$	65.13%	6.82%	6.25%	8.70%
Theo. 95/5% (dtbbpy)Ni(*o*-tol)Cl/(dtbbpy)$NiCl_2$	65.66%	6.85%	6.21%	8.26%

Theo. 93/7% (dtbbpy)Ni(*o*-tol)Cl /DCM	65.51%	6.83%	6.09%	8.79%

Table S2. Calculated and measured C, H, N and Cl results for combustion analysis for given mixtures of (dtbbpy)Ni(*o*-tol)Cl and (dtbbpy)$NiCl_2$. The possibility of DCM in the EA sample is also considered, given the presence of DCM in the atmosphere of the anaerobic glovebox atmosphere.

S2. L-edge absorption spectroscopy

Solution-phase X-ray transient absorption experiments were performed at the Ni $L_{2,3}$-edges at beamline UE52_SGM at BESSY II in the *nmTransmission* NEXAFS end station, which features 70-80 ps temporal resolution[3,4]. A colliding liquid jet with two 27 µm nozzles and an HPLC pump was used to generate a thin (~2 µm) liquid jet. The operating vacuum pressure of the chamber with the liquid jet flowing was $\sim 2 \cdot 10^{-3}$ mbar. The spent solution was frozen out in a cold trap below the chamber. Spatial overlap was first determined by visualizing the laser spot and X-ray spot on a YAG screen mounted in approximately the same plane as the sample. Time zero was determined using a solution of 20 mM $Fe(bpy)_3Cl_2$ in water, and spatial overlap was tuned on this sample via manual movement of a motorized mirror. The energy was calibrated by comparing the L_3 centroid of the ground-state spectrum to representative literature Ni(II) spectra of model compounds including Ni(II)F_2 and Ni(II) bis(diphenylbis((methlthio)methyl)borate), and the experimental spectrum was shifted accordingly[5]. The L_2-edge spectrum and transient spectra were also shifted accordingly. The sample was dissolved in DMF to generate a 20 mM solution. The solution was filtered through a 0.45 µm PTFE filter. The sample bottle was attached to a switcher connected to the HPLC pump, and bare DMF was also attached to the switcher. The bare DMF was used to generate the liquid jet at ambient pressure, and the switcher was used to switch to a sample jet once operating pressure in the sample chamber was reached. The sample bottle was backfilled with N_2 by using a N_2-filled balloon.

The sample was pumped with 350 fs pulses at 343 nm (third harmonic of 1030 nm) from a Tangerine laser (Amplitude Systèmes) triggered by the synchrotron RF cavity at a fluence of 100 mJ/cm^2 (absorbed fluence ~3 mJ/cm^2). The relative time delay between the X-rays and laser was set to 100 ps, and the monochromator and undulator were scanned at the Ni L_3 and L_2 edges and the transmitted X-ray beam was collected on an APD detector connected to a Boxcar integrator (HHFLI Zurich Instruments). For kinetic traces, the monochromator energy was set to the peak of the Ni L_3 transient and the laser delay was scanned. The time resolution of 70-80 ps is due to the X-ray pulse width of the hybrid bunch in the BESSY II fill pattern. The ground state spectra were collected without the laser and with the full X-ray flux transmitted on a GaAs detector.

The fine-tuned spatial overlap was performed on the N K-edge of $Fe(bpy)_3Cl_2$ in H_2O. The spatial overlap is performed with a remote-controlled motorized mirror. To maximize stability of the liquid jet, spatial overlap was found and then the chamber was vented to switch to the DMF/sample (switching directly between the water system and the DMF system led to rapid clogging of the liquid jet). Because the signal was several orders of magnitude smaller at the Ni L-edge than at the N K-edge in $Fe(bpy)_3Cl_2$, we did not optimize spatial overlap on the sample in DMF. While it is not expected to change significantly between the two samples, there may be slight differences in the liquid jet between water and DMF, and therefore the spatial overlap on the sample may be slightly imperfect.

The solid state spectrum of (dtbbpy)Ni(*o*-tol)Cl was collected at Beamline B07: Versatile Soft X-ray (VerSoX)[6,7] by Matthijs van Spronsen at the Diamond Light Source in total electron yield (TEY). Fig. S1 shows the solid phase data overlapped with the solution phase data. The spectra are nearly identical, indicating that the structure of the complex is similar in both the liquid and solid phase. Since the crystal

structure[1] shows a near-square-planar coordination geometry, we conclude that the triplet character of the (dtbbpy)Ni(*o*-tol)Cl complex does not arise from distortions towards tetrahedral geometry in solution, e.g. by solvent coordination at the axial positions. Due to fluctuations of the liquid jet, the L_3 and L_2-edge spectra could not be collected in a single scan and were collected separate, so their relative intensities in solution cannot be determined.

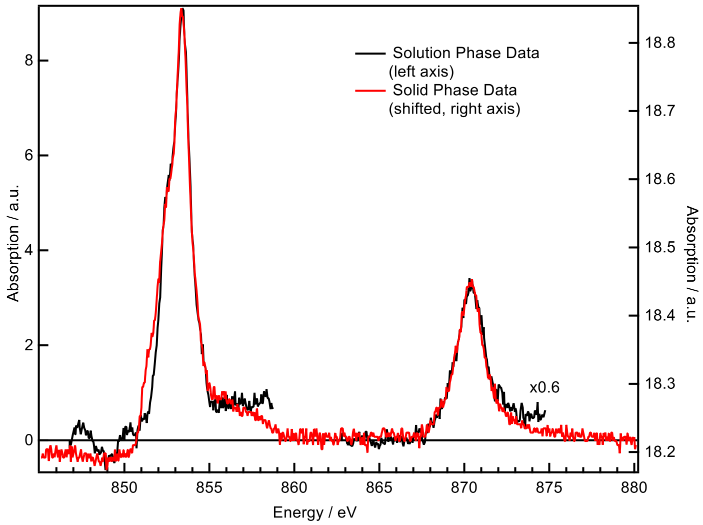

Figure S1. Comparison of the solid and solution phase L-edge spectra of (dtbbpy)Ni(*o*-tol)Cl. The solution phase L_2 edge spectrum has been arbitrarily scaled to match the solid phase L_2 edge peak intensity.

S2.1 CTM Simulations

XAS spectra were simulated with the CTM4XAS code[8]. Charge-transfer parameters were chosen based on previous literature of Ni^{2+} species[9]. The charge transfer parameters are: Δ, the energy of the $3d^9\underline{L}$ state relative to the $3d^8$ configuration; U_{dd}-U_{pd}, the energy of the secondary core-hole configuration relative to the first in terms of the Hubbard U which describe the increased 3d repulsion and decrease 3p repulsion; and the hopping integrals, $T(b_1)$, $T(a_1)$,$T(b_2)$, and T(e) for D_{4h}; and $T(e_g)$ and $T(t_{2g})$ for T_d, also called the overlap integrals, which parameterize the mixing of the configurations. The CT input parameters for all simulations (with the exception of d^9, wherein charge transfer would lead to a $3d^{10}$ state and thus no transition) were set to $\Delta = 0$, U_{dd}-$U_{pd} = 2$ eV, $T(b_1)=1$, $T(a_1)=T(b_2)=T(e)=2$. The simulation must incorporate charge transfer to have a singlet ground electronic state. Simulations in the absence of CT to model covalency led to triplet ground states. Resulting stick spectra were broadened with a 0.3 eV Gaussian function (to model the energy resolution of the beamline) and a 0.3 eV Lorentzian (to model the core-hole lifetime broadening).

In a D_{4h} ligand field, the crystal field parameters are $10D_q$ (octahedral field splitting) and additional parameters D_s and D_t. The orbitals are split according to the following: $d_{xz,yz} = -4D_q-D_s+4D_t$; $d_{xy} = = -4D_q+2D_s-D_t$; $d_{z2} = 6D_q-2D_s-6D_t$; $d_{x2-y2} = 6D_q+2D_s-D_t$. The crystal field parameters that yield the best match are $10D_q = 2.02$ eV, $D_t = 0.24$ eV, and $D_s = 0.4$ eV. The simulations of the d^7 and d^9 states were carried out with the same crystal field parameters and only the number of d-electrons was varied. The excited crystal-field ^{3}dd and ^{1}dd states were the lowest pure singlet and pure triplet states in the ground state ligand field parameters. For all states, the $3d^9\underline{L}$ contribution is ~45%.

The tetrahedral excited state did not use charge transfer parameters, and the simulation was performed with an O_h ligand field with a $10D_q$ of -1.5 eV.

We have investigated two methods of introducing covalency into the system, and we find that they both yield similar results and spectra. Instead of applying charge transfer parameters, the atomic Slater orbital pre-factor can similarly be reduced to model charge transfer. Figure S2 compares the two models, with both models reasonably simulating the low-energy shoulder. The high-energy feature is better modeled by the charge transfer model. We have chosen to use the charge transfer model in our analysis due to the better match between experiment and simulation. The semi-empirical nature of the CTM4XAS calculation means that a perfectly square-planar ligand field is assumed, which is not the case based on the crystal structure. This assumption could account for the discrepancies between theory and experiment.

Interestingly, the above crystal field and charge transfer parameters yields a mixture of ~70% singlet and ~30% triplet in the ground state of the complex. Partial triplet-state character has been shown to occur for Co2+/3+ complexes inferred by L-edge spectroscopy[10]. However, due to the phenomenological nature of the CTM4XAS method, that is expected to only yield a qualitative match to the data, the indicated spin mixture in the ground state warrants further investigations.

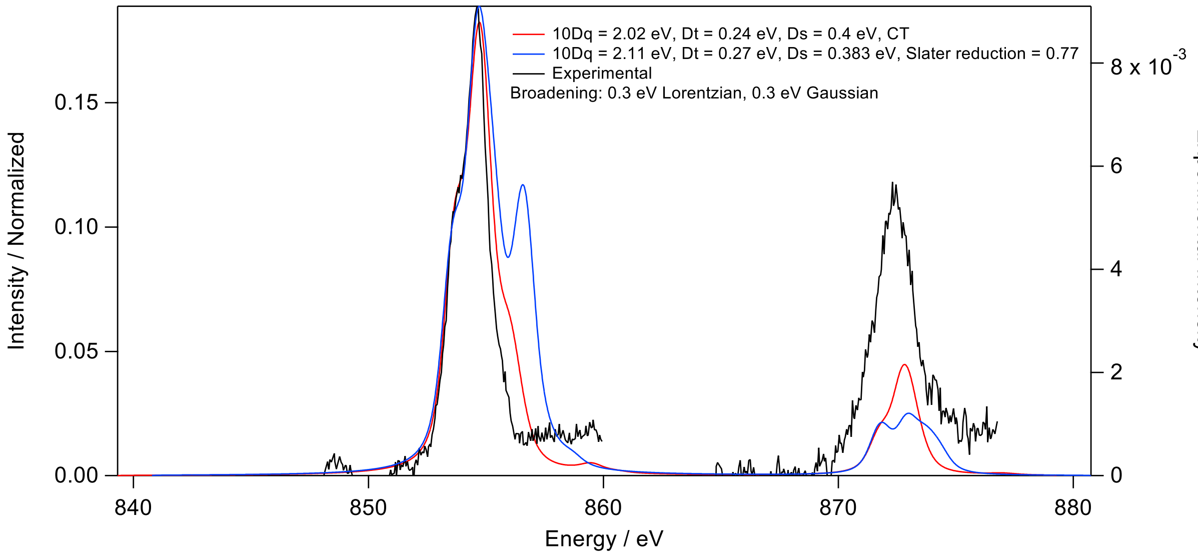

Figure S2. Comparison of addition of covalency with charge transfer (red trace) or Slater reduction (blue trace).

To show that the differences in the ground-state spectrum have relatively little effect on the assignment of the excited-state, we took the CTM4XAS parameters of NiOEP from Reference 9 (Figure S3) and computed the tetrahedral – square planar difference spectrum (Figure S4). The result is very similar to our best fit difference spectrum in the main text.

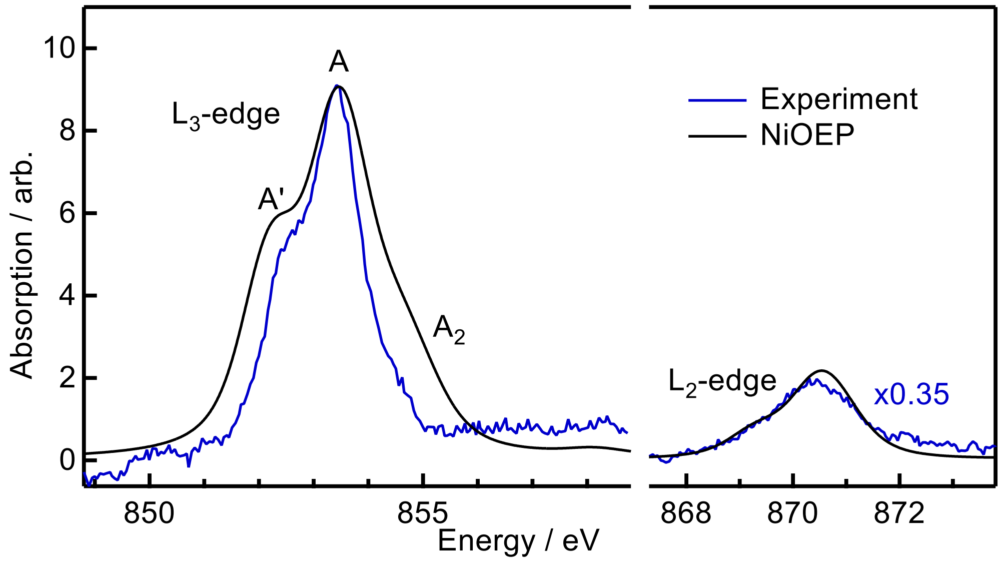

Figure S3. Experimental spectrum of (dtbbpy)Ni(o-Tol)Cl compared to the spectrum given by the NiOEP parameters from Reference 9 with a broadening of 0.35 eV Lorentzian and 0.35 eV Gaussian.

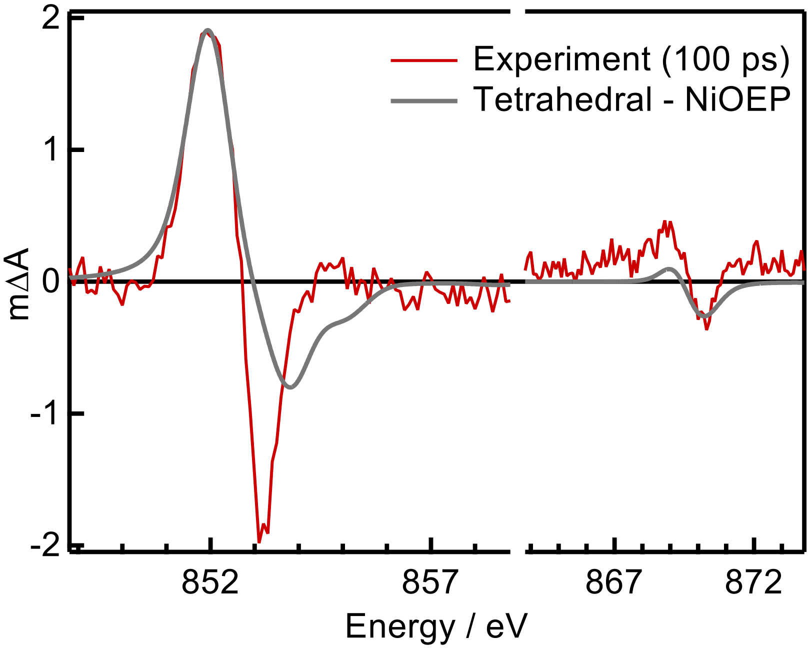

Figure S4. Tetrahedral – NiOEP simulation compared to experiment.

S3. NMR Characterization

Proton NMR was recorded at the UIUC School of Chemical Sciences NMR Lab. Chemical shifts are reported in ppm and were converted to Hz using MestreNova. NMR measurements were performed at 15 mM complex in d2-DCM dried over molecular sieves for two days. 1,3-benzodioxole purchased from Sigma was used as an internal standard without further purification. The chemical shifts are in excellent agreement with previous studies.[1] For the Evans method experiments, a capillary containing the deuterated solvent with the internal standard was flame sealed and inserted into the NMR tube. Concentration dependent measurements were performed in d2-DCM at concentrations of 10, 15, 20, 30, and 60 mM. Variable temperature Evans method measurements were performed on a 600 MHz instrument at a 10 mM sample concentration in CD_2Cl_2. A known amount of standard was added to the NMR tube and to the capillary. The sample was cooled to 188 K and was warmed up at intervals of 15 K. The temperature was allowed to equilibrate for ten minutes at each data point. The diamagnetic correction factor was applied to all of the calculated μ_{eff} values[11].

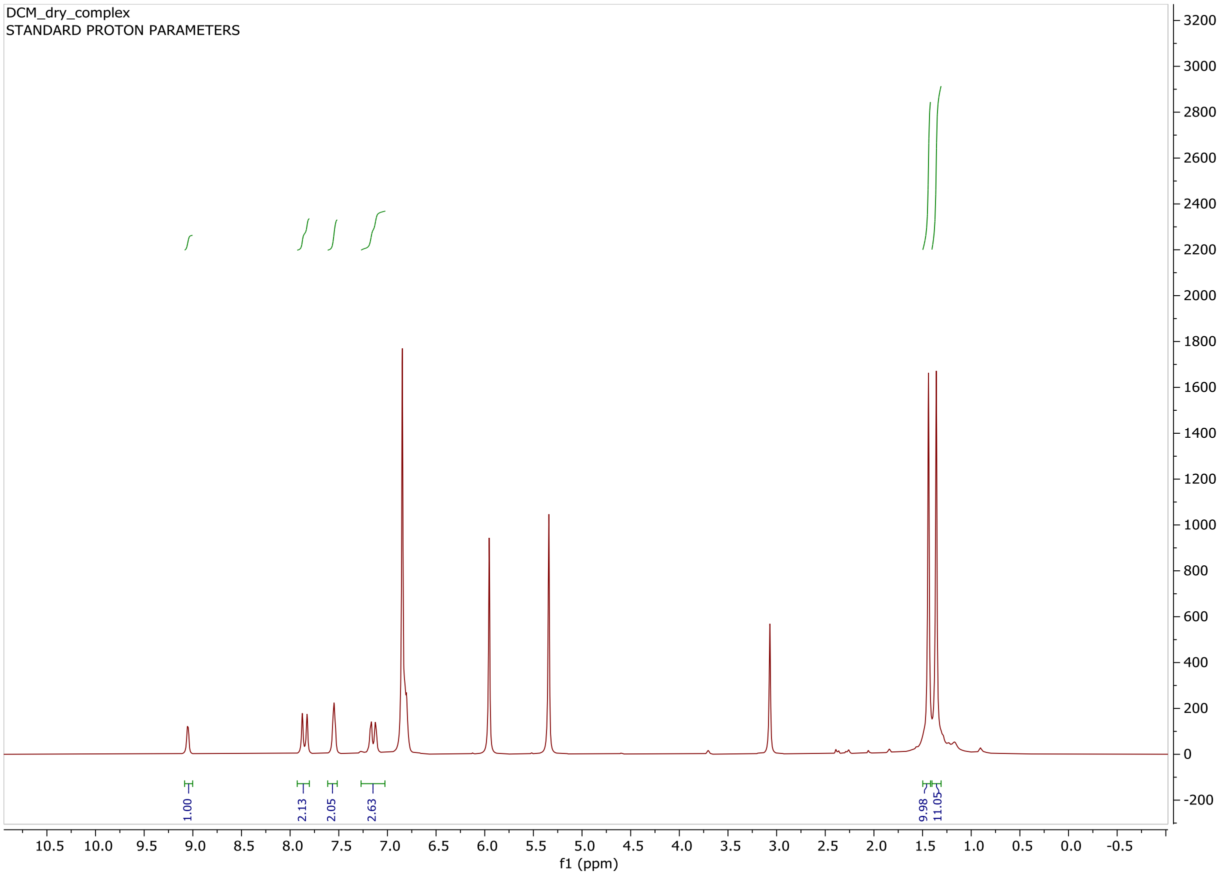

Figure S5. Proton NMR spectrum in recorded in d2-DCM with 1,3-benzodioxole added as an internal standard.

Clearly, the integrations reported herein would indicate a normal diamagnetic NMR spectrum. However, when the peaks from the complex are integrated with respect to the standard (Figure S5), the integrations of the sample peaks are much lower than the actual number of protons.

The diamagnetic purity of the complex was calculated according to the following equation:

$$P_i = \frac{AI_i}{AI_r} \times \frac{NN_r}{NN_i} \times \frac{MW_i}{MW_r} \times \frac{M_r}{M_i} \times P_r$$

Where subscripts *i* and *r* refer to the analyte and reference compound, respectively, *P* is the purity, *AI* is the absolute integral of the relevant NMR peak, *NN* is the number of nuclides, *MW* is the molar mass, and *M* is the mass of material used in grams. The reference was assumed to be 100% pure (rounded up from 99.9% purity reported by Sigma).

The peak at 5.95 ppm corresponds to aliphatic protons of benzodioxole (2H, area = 4.4) and the peak at 1.4 ppm corresponds to one of the *t*-butyl peaks of the complex (9H, area = 9). The *t*-butyl peak area was set to 9 and the benzodioxole peak area was computed by MestreNova based on this. Using these peaks and the above equation, we find that the complex in d2-DCM contains 45% diamagnetic material and the complex in d7-DMF contains 50% diamagnetic material. Although the solvents were dried over molecular sieves, there may be a residual water peak behind the *t*-butyl peak that could cause some discrepancies. This result could be due either to an inherent triplet ground-state or due to the presence of an NMR-silent, optically silent impurity.

To investigate the combustion analysis results, we spiked the (dtbbpy)Ni(*o*-Tol)Cl with 30% of (dtbbpy)$NiCl_2$ and recorded the NMR spectrum. The two species undergo a self-exchange reaction, resulting in new paramagnetic peaks.

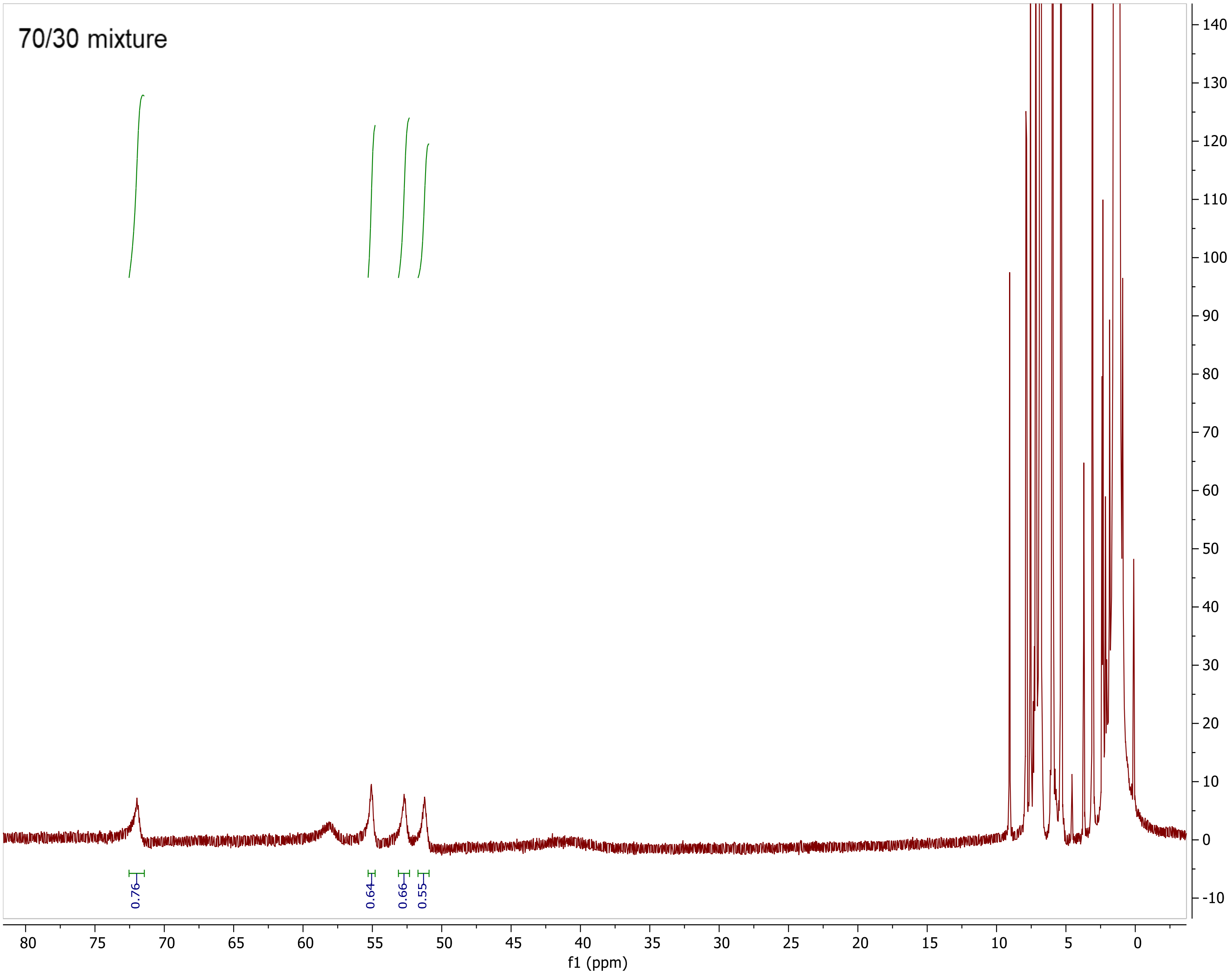

Figure S6. The NMR spectrum of a 70/30 mixture of (dtbbpy)Ni(*o*-Tol)Cl/(dtbbpy)$NiCl_2$. The four integrated peaks are unique to the mixture. The two paramagnetic peaks at 42 ppm and 57 ppm are from the (dtbbpy)$NiCl_2$ complex.

These peaks are also present in the as-prepared (dtbbpy)Ni(*o*-Tol)Cl complex.

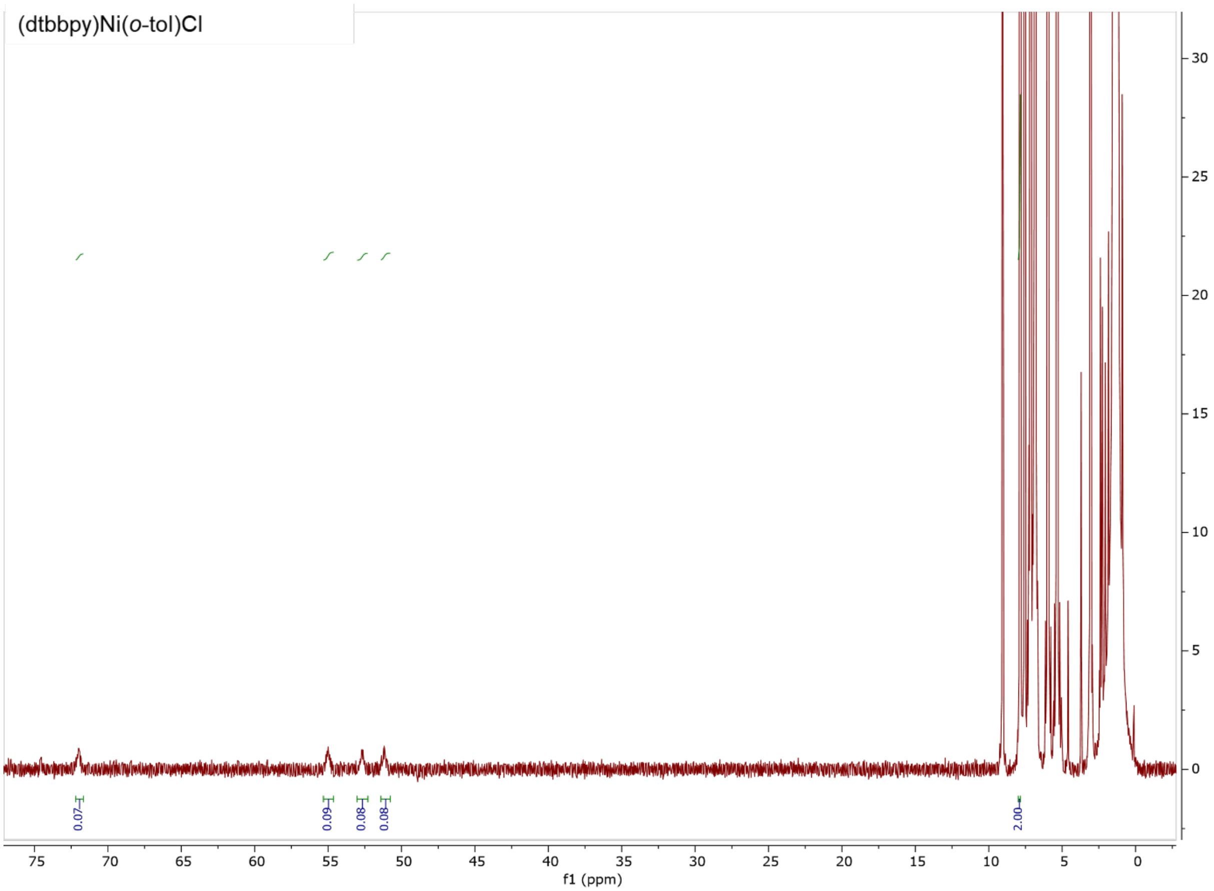

Figure S7. As-prepared (dtbbpy)Ni(*o*-Tol)Cl complex with the self-exchange paramagnetic peaks integrated.

To semi-quantitatively determine how much (dtbbpy)$NiCl_2$ is present in the as-prepared sample, we compare the integrations of the self-exchange peaks. We find that the peak integrals in the paramagnetic region of the as-prepared sample are approximately ten times smaller than in the spiked sample. Therefore, we estimate that there is approximately ten times less (dtbbpy)$NiCl_2$ in the as-prepared sample than in the spiked sample. This estimate of 3% of (dtbbpy)$NiCl_2$ in the as-prepared sample is in good agreement with our combustion analysis results, see SI Section S1.

S4. Optical absorption spectroscopy

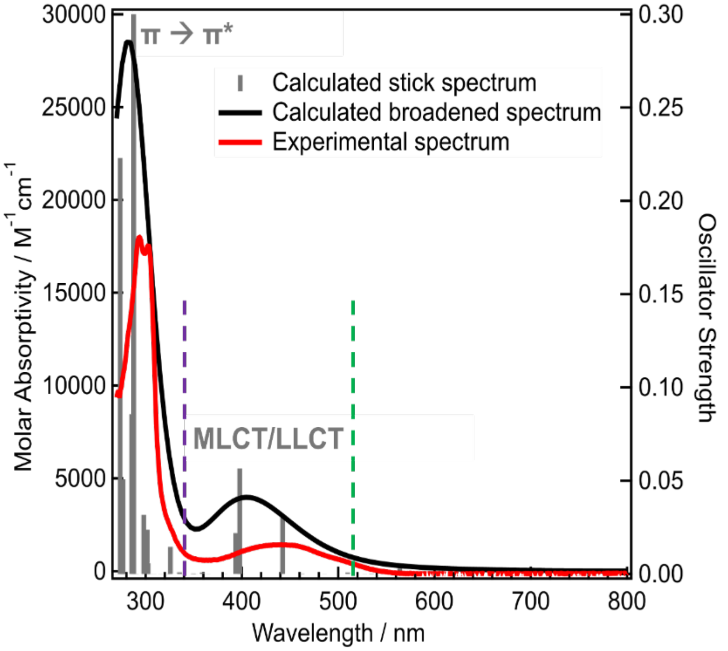

Figure S8. Experimental optical absorption spectrum and calculated spectrum (B3LYP/TZVP, PCM model, solvent = DMF) in black reported in molar absorptivity (left axis), with corresponding stick spectrum (right axis). The pump wavelength in the L-edge transient absorption experiment is denoted with the purple dashed line, and the pump wavelength in the K-edge transient absorption experiment is denoted with the green dashed line.

UV/Vis spectra were recorded on a Varian Cary 50 instrument and are reported in molar absorptivity (M^{-1} cm^{-1}) vs wavelength (nm). The UV/visible absorption spectrum of (dtbbpy)Ni(*o*-tol)Cl in dimethylformamide (DMF) is shown in Figure S8. The complex features a weak low-energy absorbance feature that is assigned to a metal-to-ligand charge transfer (MLCT) transition mixed with chloride/*o*-tolyl ligand-to-bpy ligand charge transfer (LLCT) (see Section S7). The dominant UV transition is of bpy π → π* character. The L-edge XTA experiment used a 343 nm pump wavelength (purple dashed line) which is at the very edge of this ligand-centered transition, and the K-edge XTA experiment used a 515 nm pump wavelength (green dashed line) which is at the red edge of the MLCT transition.

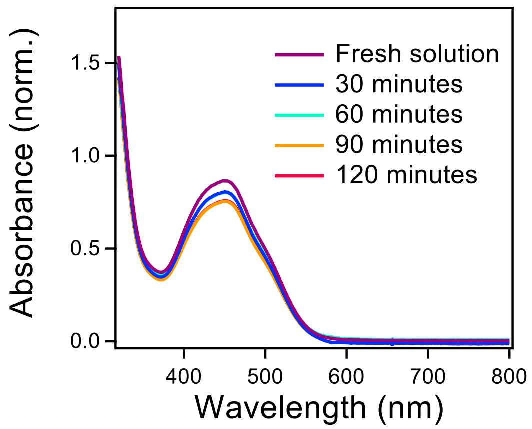

Figure S9. UV-vis stability study of (dtbbpy)Ni(o-tol)Cl in DMF over the course of two hours. The traces are normalized to 330 nm.

The degradation of the complex in DMF under nitrogen and light (conditions used for L-edge XTA experiments) is slow, with a gradual loss of the MLCT intensity (Figure S9). The intensity loss over the course of two hours is ~10%. Most of this loss occurs within the first 90 minutes and then degradation slows, so we assume that the degradation in the course of one night of XTA measurement is minimal. The initial fast degradation could be due to conditions such as residual water contained in the solvent, imperfectly dry glassware, or air exposure when preparing the solution. One solution was measured for no more than six hours at a time, and X-ray/laser damage were not factors for the L-edge experiments due to the one-way flowing system.

A 1 kHz Ti:sapphire regenerative amplifier (Spitfire, Spectra-Physics) pumped by an Empower and seeded by a MaiTai (Spectra-Physics) generated 800 nm pulses of approximately 100 fs with 2.5 W total output power. The 800 nm pulse was aligned into a TOPAS Twins (Light Conversion) and 10% of the pulse was aligned out of the TOPAS to generate the probe beam. The remaining 90% was used to generate an IR signal which was then quadrupled in a NIRUVis (Light Conversion) to generate the 343 nm pump beam. The pump pulse was chopped at 500 Hz and was focused to a spot size of 90 μm FWHM using a spherical mirror. The polarization of the pump beam was not controlled.

The remaining 800 nm light was passed through an optical retroreflector mounted on a motorized delay stage and passed through a λ/2 waveplate to enable magic angle experiments. The 800 nm light was focused into a translating 3 mm crystalline CaF_2 to generate the white light continuum from 300 to 700 nm. The white light is then sent through a filter to remove the remaining 800 nm light. The probe was then focused onto the sample using an off-axis parabolic mirror to a spot size of 5 μm FWHM. The transmitted probe beam was focused into a fiber and directed into a CMOS transient absorption spectrometer (Ultrafast Systems), and data was collected shot-to-shot. Samples were prepared in a N_2-filled glovebox and concentrations were tuned to give an optical density of ~0.5 at the pump wavelength. The samples were prepared in 2 mm quartz cuvettes (Starna and FireflySci). Samples were stirred during experiments using a magnetic stirrer. Each data set is the average of at least 30 scans with 0.2 second averaging at each delay. The cross-phase modulation was monitored using a cuvette of the bare solvent and was fitted to a polynomial to correct for temporal chirp. The data sets were background subtracted by subtracting the averaged transient before the cross-phase modulation signal. UV-vis spectra of the samples before and after transient absorption measurements were collected to monitor for sample damage.

Global fitting was performed in the program *IgorPro*. A three-component sequential decay was used on the basis of literature precedent. The differential equation for the decay was solved in *Mathematica*, and then an *IgorPro* fit model was written. The exponentials were convoluted with a Gaussian function to fit time-zero and the instrument response function (IRF). The kinetics and spectral features are relatively insensitive to solvent with the exception of shifts of the GSB, as expected given the solvatochromism seen in the ground state spectra.

OTA experiments were performed in DMF, THF and toluene solvents. However, due to the large two-photon absorption cross-section of toluene at 343 nm, we were unable to eliminate spurious solvent signals and can only use this data set qualitatively to confirm that the signal is the same shape as in the other solvents. We were also able to perform the measurement in acetonitrile, but due to the instability of the complex in this solvent, we were only able to collect ~5 scans before the complex and stock solution fully degraded.

Since the ultrafast L- and K-edge XAS experiments were performed in DMF, we present the OTA results in this solvent in the next Section.

S4.1 OTA in DMF

The OTA spectrum is characterized by a ground state bleach (GSB) of the MLCT transition centered at 450 nm, an excited state absorption (ESA) in the UV, and a second ESA in the visible (Figure S10). We see three general timescales of spectral evolution in the signal: first, in the sub-ps regime we see a decay of the bleach and a slight decay in the visible ESA. Second, in the 5-10 ps regime, we see a complete loss of the visible ESA and narrowing of the GSB. Finally, the remainder of the data exhibits a slow decay of the entire signal (Figure S10D). This can be globally fitted to a three-exponential decay to yield time constants of τ_1=0.6 ± 0.02 ps, τ_2=6 ± 0.2 ps, and τ_3=3090 ± 50 ps (Figure S11). These are in good agreement with the literature and the first time scale is assigned to the decay of the initially-excited MLCT state, the second time scale to vibrational relaxation or inter-system crossing within the MLCT state, and the third time scale to a long-lived photoexcited state that cannot be unambiguously determined using optical techniques[1,12].

The transient spectrum of (dtbbpy)Ni(*o*-tol)Cl in DMF following photoexcitation at 343 nm resembles the spectra in benzene and THF reported in the literature with λ_{ex}>400 nm[1,12] indicating that 343 nm photoexcitation populates the same excited state as higher wavelength photoexcitation (Figure S12).

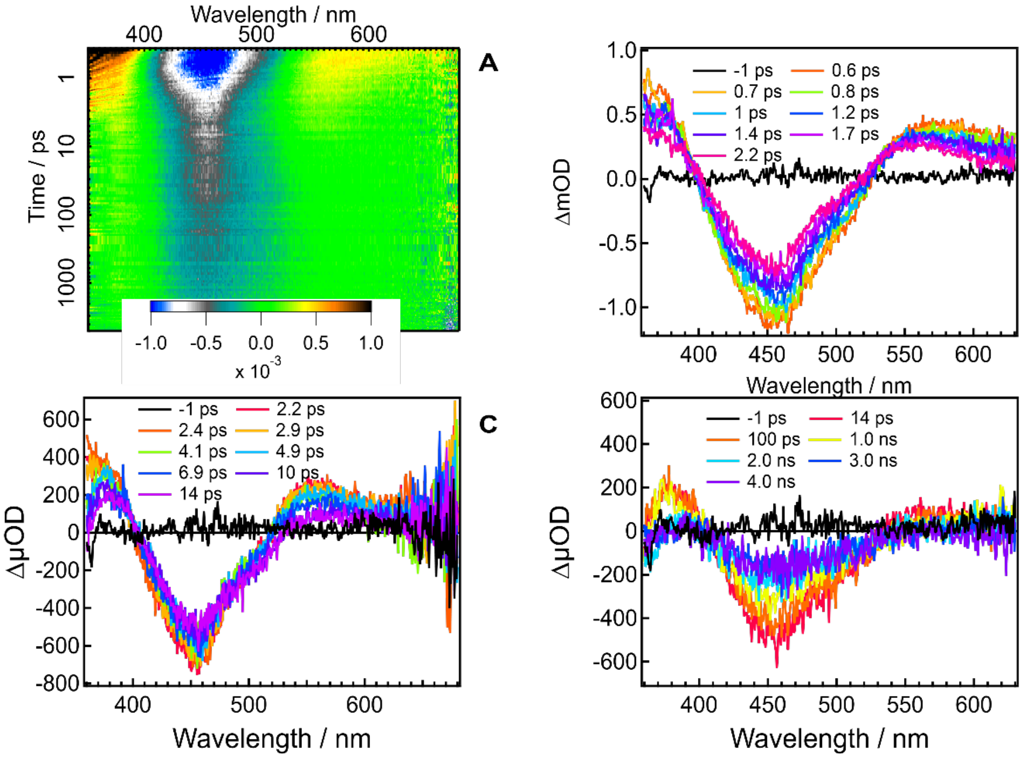

Figure S10. (A) 2D OTA spectrum of (dtbbpy)Ni(o-tol)Cl in DMF following photoexcitation at 343 nm; (B) Spectral traces at selected early time delays showing GSB decay and visible ESA decay; (C) Spectral traces between 2 and 15 ps showing GSB narrowing and decay of the visible ESA; (D) Spectral traces from 15-4000 ps showing slow decay of the entire signal.

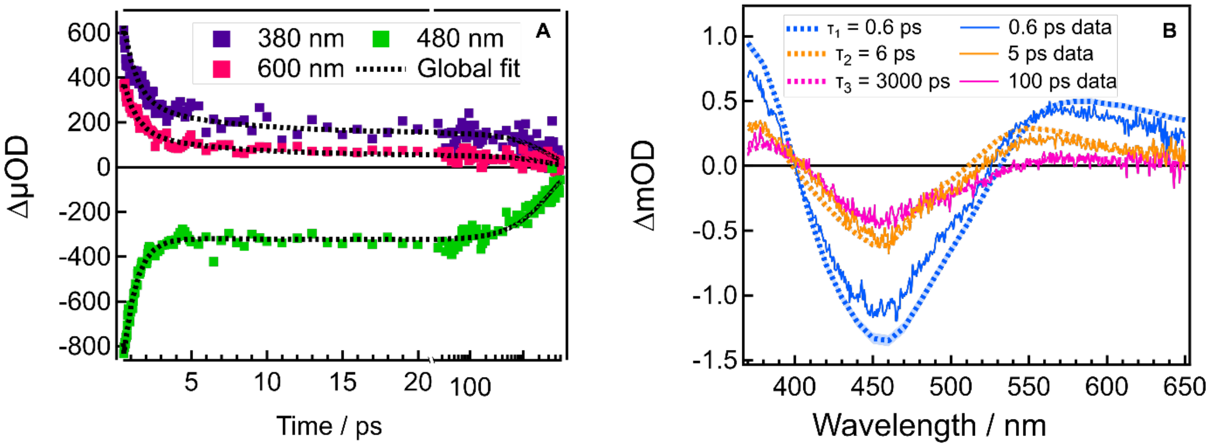

Figure S11. (A) Kinetic traces at selected wavelengths (colored squares) with corresponding global fit (dashed lines, 380 nm → UV ESA; 480 nm → GSB; 600 nm → visible ESA); (B) Species Associated Decay Spectra (SADS, dashed lines) along with spectral traces at selected time delays (solid lines) to show the match between data and fit.

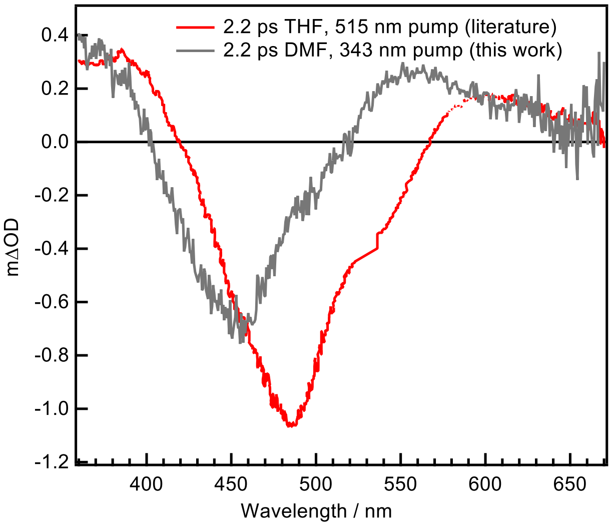

Figure S12. Comparison of the transient spectrum following 515 nm photoexcitation in THF in red (adapted from Ref. 1) and the transient spectrum following 343 nm photoexcitation in DMF in gray. The spectra are identical except for the solvatochromic shift in the ground-state bleach between the two solvents.

S5. K-edge X-ray absorption spectroscopy

Ground- and excited-state K-edge measurements were collected at beamline 11-ID-D of the Advanced Photon Source. The sample was pumped with a 515 nm, 100 fs pulse generated in an OPA pumped by a Ti:Sapphire laser operating at 3 kHz. The incident fluence was approximately 30 mJ/cm^2. An approximately 5 mM sample solution in DMF was flowed through a 760 μm cylindrical jet and the total fluorescence was collected using APDs mounted at 90° relative to the incoming beam. A combination of Soller slits and Co foil were used to minimize the detection of elastically scattered X-rays on the APDs. The energy was calibrated with a Ni foil measurement. *Ab initio* calculations of XAS spectra were performed with the FDMNES code[13]. The FDMNES code was chosen for calculations of the XAS spectra because the muffin-tin potential, wherein the interstitial potential regions is flat and set to zero, is avoided. The input structure for the ground-state was the DFT-optimized square planar structure with the *t*-butyl groups truncated and replaced with protons. A cluster size of 6Å was used to encompass all atoms in the calculation. To simulate the excited-state, the optimized triplet ground state (tetrahedral) with the *t*-butyl groups truncated was used as the input structure, again with a cluster size of 6Å to encompass all atoms in the calculation. Screening of the 4p electron was investigated with the keyword "screening" but the changes to the spectrum were minimal and so the screening keyword was not used. The keyword "quadrupole" was used to calculate 1s→3d transitions. The calculated XAS is convoluted with an energy-dependent arctan function implemented in FDMNES to account for core-hole lifetime broadening to yield the K-edge spectrum. Output K-edge spectra are then normalized to one.

The K-edge spectrum was used to confirm the Ni(II) oxidation state of the complex. A comparison with a Ni(II) porphyrin spectrum measured at the same beamline shows the 1s→$4p_z$ peak and white line at the same energy positions as those transitions in the complex as well as a good general match to the shape of the spectrum[15] (Figure S13A, black trace), confirming that the complex is Ni(II) oxidation state and expected d^8 electron configuration and square planar geometry. The FDMNES spectrum vastly overestimates the intensity of the 1s → $4p_z$ transition, but yields a good match in energy for the peaks (Figure S13A, gray trace). This has been previously observed with FDMNES simulations on Ni complexes[16]. We also note that a recent XAS study on a series of Ni(II/IIII/IV) compounds confirms this result[17]. This study compares the pre-edge energies of various Ni complexes and finds that Ni(II) species exhibit one pre-edge transition and one or two rising-edge transitions, whereas the rising-edge transition is absent in Ni(III) species. Our experimental spectrum contains one pre-edge transition and one rising-edge transition, which is consistent with a Ni(II) center.

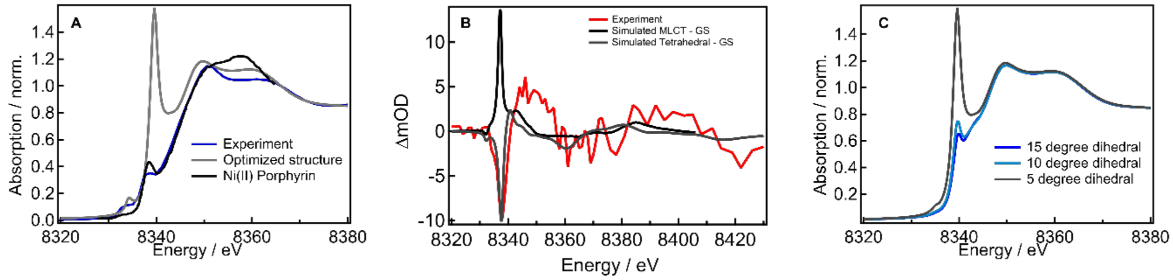

Figure S13. (A) Experimental K-edge XANES spectrum of (dtbbpy)Ni(o-tol)Cl (blue trace) overlaid with the spectrum of Ni(II) TMP in toluene adapted from Reference 14. The FDMNES simulation on the geometry-optimized complex is shown in black; (B) Comparison of the simulated transient using the "MLCT" geometry (elongated Ni-ligand bonds) in black and the tetrahedral transient (gray trace).

To simulate the MLCT spectrum, the Ni-ligand bonds were elongated by 0.07 Å in the optimized square planar structure and the FDMNES simulation was run (Figure S13B). The transient simulated spectra were scaled by 0.35 to match the experiment. Crystal structures of Ni(III) species contain longer average Ni-N bond lengths than Ni(II) species of the same ligand[14], and since the MLCT state would generate a Ni(III) species, this is the expected structural change. Clearly, the simulation for the MLCT state does not match the experiment since the bond elongation causes the 1s → $4p_z$ intensity to increase. The FDMNES calculation was also used to examine the impact of dihedral angle on the XAS spectrum (Figure S13C). As the dihedral angle between the bipyridine and ArCl planes increases (e.g. tending towards tetrahedral), the intensity of the 1s → $4p_z$ transition changes drastically . The simulated spectra were normalized to one by taking the output of the FDMNES file and scaling the spectrum by a factor of 40.

S6. Density Functional Theory Calculations

DFT optimizations were performed in Gaussian 16 with the B3LYP functional and TZVP basis set in the gas phase with an implicit solvent (DMF) using the PCM model. The input structure was the crystal structure (non-disorder model) and optimization/frequency calculations were performed on a singlet state and a triplet state to ensure no imaginary frequencies were obtained. Consistently, the singlet state optimizes to a square planar structure and the triplet state optimizes to a tetrahedral structure. The optimized triplet structure has a computed energy of +0.55 eV (12.8 kcal mol^{-1}) relative to the singlet (ground) state. TDDFT calculations were performed in Gaussian 16 with the B3LYP basis set and TZVP functional with the PCM implicit solvent model in DMF, as this is what has been shown in the literature to give the best match between theory and experiment for this molecule. Molecular orbital diagrams and natural transition orbitals were visualized with Chemissian using an isovalue of 0.02. The extinction coefficient in M^{-1} cm^{-1} of the calculated spectrum (Figure S8) was computed by broadening the TDDFT computed oscillator strength sticks with Gaussian line shapes according to the following equation[18]:

$$\varepsilon(\tilde{\nu}) = \sum_{i=1}^{n}\left(1.306274 \cdot 10^{8} \cdot \frac{f_i}{\sigma} \exp\left[-\left(\frac{\tilde{\nu} - \tilde{\nu}_i}{\sigma}\right)^2\right]\right)$$

The results reported herein are an excellent match to literature.

Optimized Structures and Cartesian Coordinates

Square planar singlet

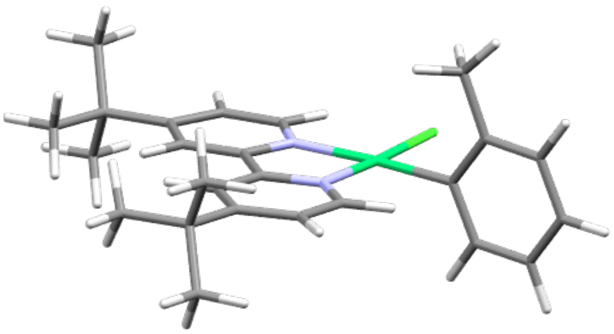

Figure S14. Optimized structure of the singlet ground-state structure of (dtbbpy)Ni(*o*-tol)Cl using B3LYP/TZVP functional/basis set combination. Implicit solvation of DMF was included using the polarizable continuum model.

Cartesian coordinates of the optimized singlet ground-state structure and thermal data:

Lowest vibrational frequency:

Zero-point correction: 0.501548 Hartrees

Thermal correction to Energy: 0.532150 Hartrees

Thermal correction to Enthalpy: 0.533094 Hartrees

Thermal correction to Gibbs' Free Energy: 0.438665 Hartrees

Sum of electronic and thermal Energies: -3049.393632 Hartrees

Sum of electronic and thermal Enthalpies: -3049.392688 Hartrees

Sum of electronic and thermal Free Energies: -3049.487117 Hartrees

B3LYP Energy: -3049.925782 Hartrees

Lowest vibrational frequency: +13.89 cm^{-1}

Ni	-1.2470000	-1.3370000	-0.0980000
N	0.7590000	-1.5130000	-0.0560000
Cl	-1.6340000	-3.5450000	-0.0610000
C	1.4840000	-2.6380000	-0.0520000
H	0.9170000	-3.5580000	-0.0630000
C	2.8670000	-2.6360000	-0.0350000
H	3.3800000	-3.5870000	-0.0330000
N	-0.7990000	0.5560000	-0.0930000
C	3.5720000	-1.4270000	-0.0200000
C	2.8020000	-0.2650000	-0.0260000
H	3.2820000	0.7000000	-0.0170000
C	1.4120000	-0.3340000	-0.0440000
C	0.9900000	2.1550000	-0.0190000
H	2.0530000	2.3270000	0.0160000
C	0.5250000	0.8430000	-0.0510000
C	-1.2520000	2.9080000	-0.0750000
H	-2.0150000	3.6750000	-0.0870000
C	0.1070000	3.2320000	-0.0280000
C	-1.6580000	1.5880000	-0.1050000

H	-2.7050000	1.3320000	-0.1410000
C	5.1030000	-1.4150000	-0.0010000
C	5.6270000	-2.1360000	-1.2640000
H	5.2920000	-3.1730000	-1.3090000
H	6.7190000	-2.1370000	-1.2610000
H	5.2900000	-1.6300000	-2.1710000
C	5.5960000	-2.1600000	1.2590000
H	5.2350000	-1.6740000	2.1680000
H	6.6880000	-2.1590000	1.2840000
H	5.2640000	-3.1990000	1.2750000
C	5.6760000	0.0110000	0.0200000
H	5.3840000	0.5810000	-0.8650000
H	6.7660000	-0.0400000	0.0330000
H	5.3610000	0.5640000	0.9070000
C	0.5660000	4.6920000	0.0120000
C	0.0560000	5.4140000	-1.2550000
H	-1.0320000	5.3980000	-1.3230000
H	0.4610000	4.9540000	-2.1580000
H	0.3740000	6.4590000	-1.2350000
C	2.0960000	4.8210000	0.0660000
H	2.5130000	4.3520000	0.9600000
H	2.3680000	5.8770000	0.0920000
H	2.5740000	4.3800000	-0.8110000
C	-0.0300000	5.3690000	1.2660000
H	-1.1200000	5.3550000	1.2580000
H	0.3100000	4.8740000	2.1780000
H	0.2900000	6.4130000	1.3070000
C	-5.3060000	-0.6330000	0.8090000
C	-3.9360000	-0.8830000	0.9550000
C	-3.1340000	-1.0400000	-0.1920000

C	-3.7440000	-0.9460000	-1.4470000
C	-5.1110000	-0.6970000	-1.5850000
C	-5.8980000	-0.5380000	-0.4490000
H	-5.9190000	-0.5130000	1.6970000
H	-3.1490000	-1.0700000	-2.3470000
H	-5.5550000	-0.6290000	-2.5720000
H	-6.9600000	-0.3440000	-0.5370000
C	-3.3390000	-0.9750000	2.3400000
H	-2.8270000	-1.9300000	2.4820000
H	-2.5940000	-0.1910000	2.5050000
H	-4.1050000	-0.8780000	3.1120000

Tetrahedral triplet

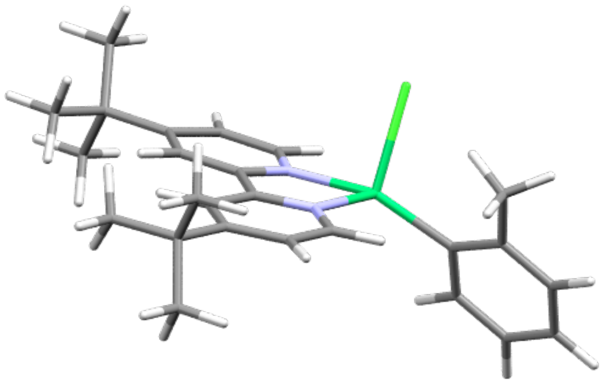

Figure S15. Optimized structure of the triplet tetrahedral excited-state of (dtbbpy)Ni(*o*-tol)Cl using B3LYP/TZVP functional/basis set combination. Implicit solvation of DMF was included using the polarizable continuum model.

Cartesian coordinates of the optimized singlet ground-state structure and thermal data:

Lowest vibrational frequency:

Zero-point correction: 0.500589 Hartrees

Thermal correction to Energy: 0.531719 Hartrees

Thermal correction to Enthalpy: 0.532664 Hartrees

Thermal correction to Gibbs' Free Energy: 0.435483 Hartrees

Sum of electronic and thermal Energies: -3049.381722 Hartrees

Sum of electronic and thermal Enthalpies: -3049.380778 Hartrees

Sum of electronic and thermal Free Energies: -3049.477959 Hartrees

B3LYP Energy: -3049.913442 Hartrees

Lowest vibrational frequency: +17.38 cm^{-1}

Ni	-1.5310000	-1.0770000	0.1940000
N	0.5000000	-1.5570000	-0.0800000
Cl	-1.4920000	-1.6840000	2.5000000
C	1.0070000	-2.7920000	-0.1440000
H	0.2920000	-3.6050000	-0.1360000
C	2.3650000	-3.0390000	-0.2110000
H	2.7030000	-4.0650000	-0.2650000
N	-0.6490000	0.8040000	0.1060000
C	3.2710000	-1.9730000	-0.1990000
C	2.7240000	-0.6920000	-0.1300000
H	3.3710000	0.1700000	-0.1110000
C	1.3430000	-0.5110000	-0.0780000
C	1.3940000	2.0230000	-0.1060000
H	2.4660000	2.0000000	-0.2110000
C	0.6920000	0.8230000	-0.0200000
C	-0.6680000	3.1910000	0.0630000
H	-1.2680000	4.0890000	0.0990000
C	0.7250000	3.2460000	-0.0600000
C	-1.3070000	1.9670000	0.1400000
H	-2.3820000	1.9020000	0.2280000
C	4.7800000	-2.2340000	-0.2590000
C	5.1080000	-2.9520000	-1.5870000
H	4.5860000	-3.9070000	-1.6720000
H	6.1800000	-3.1510000	-1.6420000
H	4.8320000	-2.3350000	-2.4440000
C	5.1830000	-3.1360000	0.9290000
H	4.9480000	-2.6570000	1.8820000
H	6.2590000	-3.3220000	0.8990000

H	4.6770000	-4.1020000	0.9000000
C	5.6010000	-0.9370000	-0.1870000
H	5.3850000	-0.2700000	-1.0240000
H	6.6630000	-1.1820000	-0.2290000
H	5.4240000	-0.3940000	0.7430000
C	1.4460000	4.5940000	-0.1510000
C	0.9470000	5.3450000	-1.4060000
H	-0.1280000	5.5270000	-1.3710000
H	1.1660000	4.7790000	-2.3140000
H	1.4480000	6.3120000	-1.4790000
C	2.9710000	4.4350000	-0.2500000
H	3.3850000	3.9280000	0.6240000
H	3.4330000	5.4220000	-0.3060000
H	3.2670000	3.8830000	-1.1440000
C	1.1210000	5.4260000	1.1100000
H	0.0520000	5.6180000	1.2080000
H	1.4580000	4.9140000	2.0140000
H	1.6300000	6.3900000	1.0550000
C	-5.8350000	-0.4440000	-0.3660000
C	-4.5680000	-0.3780000	0.2300000
C	-3.4520000	-0.9750000	-0.3980000
C	-3.6920000	-1.6190000	-1.6290000
C	-4.9530000	-1.6820000	-2.2230000
C	-6.0360000	-1.0890000	-1.5830000
H	-6.6810000	0.0160000	0.1350000
H	-2.8680000	-2.0950000	-2.1540000
H	-5.0870000	-2.1920000	-3.1710000
H	-7.0260000	-1.1290000	-2.0220000
C	-4.4420000	0.3670000	1.5410000
H	-3.5430000	0.0690000	2.0780000

H	-4.3960000	1.4500000	1.3780000
H	-5.3060000	0.1870000	2.1850000

The Natural Transitions Orbitals (NTO) from the TDDFT calculation in DMF for the relevant intense transitions are shown below. Importantly, the visible transition at 440 nm is predominantly a metal-to-bpy ligand charge transfer (MLCT) transition, with some chloride ligand-to-bpy ligand charge transfer (LLCT) character (Fig. S16). At slightly higher energy (400 nm), there is another metal-to-bpy ligand charge transfer transition and chloride/tolyl ligand-to-bpy ligand charge transfer (Fig. S17) These two overlapping transitions form the visible MLCT band in the UV/Vis spectrum. The dominant transition in the UV region occurs at 290 nm, and is an intra-bpy ligand charge transfer transition (ILCT) (Fig. S18). The pump wavelength of 343 nm excited the red edge of the ILCT transition, but based on the OTA spectra, this is insufficient energy to populate the ILCT and thus directly populates the MLCT.

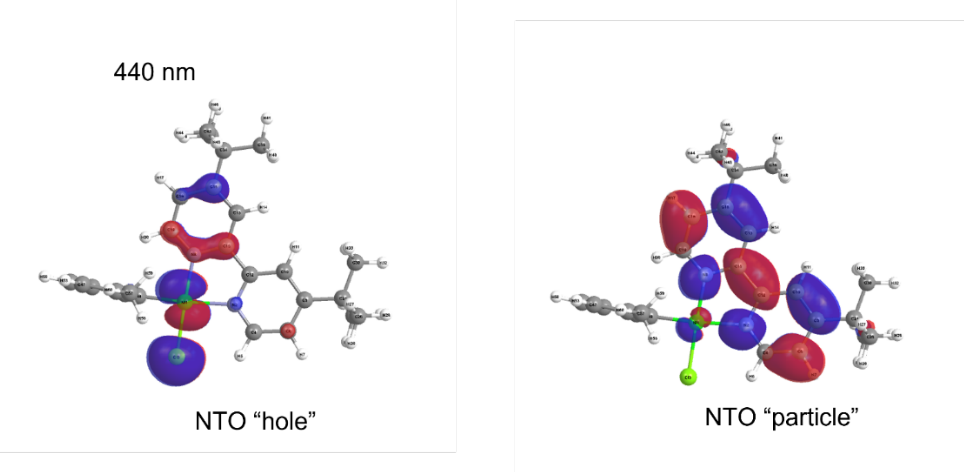

Figure S16. Natural transition orbitals for the MLCT/LLCT transition centered at 440 nm. The oscillator strength of this transition is 0.032.

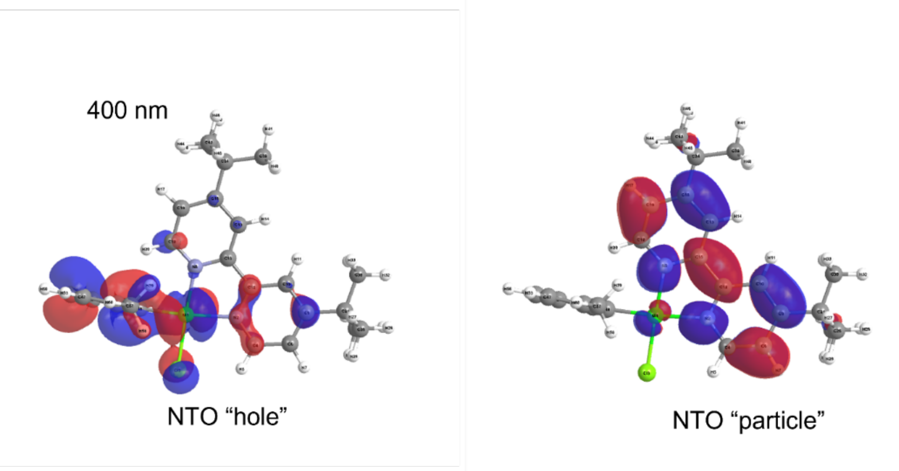

Figure S17. Natural transition orbitals for the MLCT/LLCT transition centered at 400 nm. The oscillator strength of this transition is 0.057.

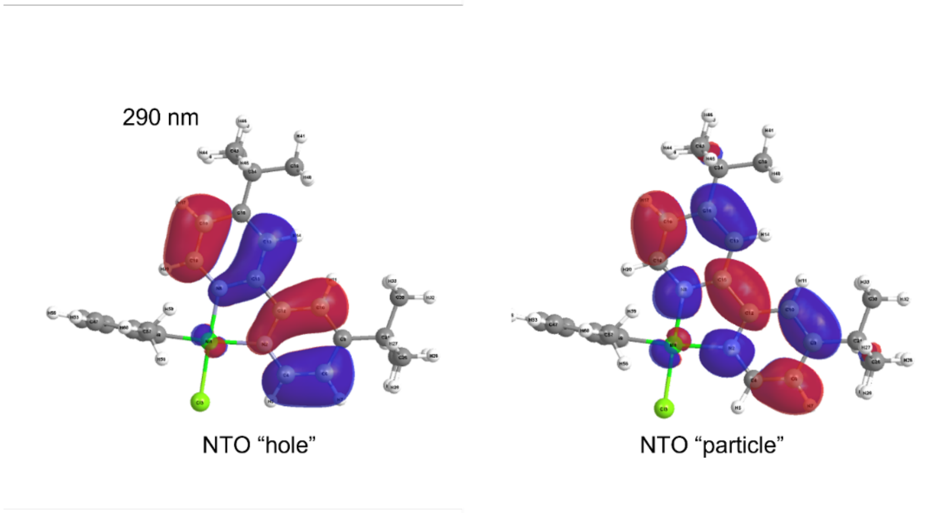

Figure S18. Natural transition orbitals for the intense ILCT transition centered at 290 nm. The oscillator strength of this transition is 0.310.

S7. References

(1) Shields, B. J.; Kudisch, B.; Scholes, G. D.; Doyle, A. G. Long-Lived Charge-Transfer States of Nickel(II) Aryl Halide Complexes Facilitate Bimolecular Photoinduced Electron Transfer. *J. Am. Chem. Soc.* **2018**, *140*, 3035–3039.

(2) E. H. Kuveke, R.; Barwise, L.; van Ingen, Y.; Vashisth, K.; Roberts, N.; S. Chitnis, S.; L. Dutton, J.; D. Martin, C.; L. Melen, R. An International Study Evaluating Elemental Analysis. *ACS Cent. Sci.* **2022**, *8*, 855–863.

(3) Fondell, M.; Eckert, S.; Jay, R. M.; Weniger, C.; Quevedo, W.; Niskanen, J.; Kennedy, B.; Sorgenfrei, F.; Schick, D.; Giangrisostomi, E.; Ovsyannikov, R.; Adamczyk, K.; Huse, N.; Wernet, P.; Mitzner, R.; Föhlisch, A. Time-Resolved Soft X-Ray Absorption Spectroscopy in Transmission Mode on Liquids at MHz Repetition Rates. *Struct. Dyn.* **2017**, *4*, 054902.

(4) Miedema, P. S.; Quevedo, W.; Fondell, M. The Variable Polarization Undulator Beamline UE52 SGM at BESSY II. *JLSRF* **2016**, *2*, 1–4.

(5) Wang, H.; Ralston, C. Y.; Patil, D. S.; Jones, R. M.; Gu, W.; Verhagen, M.; Adams, M.; Ge, P.; Riordan, C.; Marganian, C. A.; Mascharak, P.; Kovacs, J.; Miller, C. G.; Collins, T. J.; Brooker, S.; Croucher, P. D.; Wang, K.; Stiefel, E. I.; Cramer, S. P. Nickel L-Edge Soft X-Ray Spectroscopy of Nickel-Iron Hydrogenases and Model Compounds - Evidence for High-Spin Nickel(II) in the Active Enzyme. *J. Am. Chem. Soc.* **2000**, *122*, 10544–10552.

(6) Grinter, D. C.; Venturini, F.; Ferrer, P.; van Spronsen, M. A.; Arrigo, R.; Quevedo Garzon, W.; Roy, K.; Large, A. I.; Kumar, S.; Held, G. The Versatile Soft X-Ray (VerSoX) Beamline at Diamond Light Source. *Synchrotron Radiat. News* **2022**, *35*, 39–47.

(7) Held, G.; Venturini, F.; Grinter, D. C.; Ferrer, P.; Arrigo, R.; Deacon, L.; Garzon, W. Q.; Roy, K.; Large, A.; Stephens, C.; Watts, A.; Larkin, P.; Hand, M.; Wang, H.; Pratt, L.; Mudd, J. J.; Richardson, T.; Patel, S.; Hillman, M.; Scott, S. Ambient-Pressure Endstation of the Versatile Soft X-Ray (VerSoX) Beamline at Diamond Light Source. *J. Synchrotron Radiat.* **2020**, *27*, 1153–1166.

(8) Stavitski, E.; de Groot, F. M. F. The CTM4XAS Program for EELS and XAS Spectral Shape Analysis of Transition Metal L Edges. *Micron* **2010**, *41*, 687–694.

(9) Ryland, E. S.; Zhang, K.; Vura-Weis, J. Sub-100 Fs Intersystem Crossing to a Metal-Centered Triplet in Ni(II)OEP Observed with M-Edge XANES. *J. Phys. Chem. A* **2019**, *123*, 5214–5222.

(10) Sreekantan Nair Lalithambika, S.; Golnak, R.; Winter, B.; Atak, K. Electronic Structure of Aqueous [Co(Bpy) 3] 2+/3+ Electron Mediators. *Inorg. Chem.* **2019**, *58*, 4731–4740.

(11) Bain, G. A.; Berry, J. F. Diamagnetic Corrections and Pascal's Constants. *J. Chem. Educ.* **2008**,

85, 532–536.

(12) Ting, S. I.; Garakyaraghi, S.; Taliaferro, C. M.; Shields, B. J.; Scholes, G. D.; Castellano, F. N.; Doyle, A. G. 3 D-d Excited States of Ni(II) Complexes Relevant to Photoredox Catalysis: Spectroscopic Identification and Mechanistic Implications. *J. Am. Chem. Soc.* **2020**, *142*, 5800–5810.

(13) Joly, Y. Finite-Difference Method for the Calculation of Low-Energy-Electron Diffraction. *Phys. Rev. Lett.* **1992**, *68*, 950–953.

(14) Magallón, C.; Griego, L.; Hu, C. H.; Company, A.; Ribas, X.; Mirica, L. M. Organometallic Ni(Ii), Ni(Iii), and Ni(Iv) Complexes Relevant to Carbon-Carbon and Carbon-Oxygen Bond Formation Reactions. *Inorg. Chem. Front.* **2022**, *9*, 1016–1022.

(15) Shelby, M. L.; Mara, M. W.; Chen, L. X. New Insight into Metalloporphyrin Excited State Structures and Axial Ligand Binding from X-Ray Transient Absorption Spectroscopic Studies. *Coord. Chem. Rev.* **2014**, *277*, 291–299.

(16) Glover, J. L.; Chantler, C. T.; Soldatov, A. V.; Smolentsev, G.; Feiters, M. C. Theoretical XANES Study of the Activated Nickel (t-Amylisocyanide) Molecule. *AIP Conf. Proc.* **2007**, *882*, 625–627.

(17) DiMucci, I. M.; Titus, C. J.; Nordlund, D.; Bour, J. R.; Chong, E.; Grigas, D. P.; Hu, C. H.; Kosobokov, M. D.; Martin, C. D.; Mirica, L. M.; Nebra, N.; Vicic, D. A.; Yorks, L. L.; Yruegas, S.; MacMillan, S. N.; Shearer, J.; Lancaster, K. M. Scrutinizing Formally NiIV Centers through the Lenses of Core Spectroscopy, Molecular Orbital Theory, and Valence Bond Theory. *Chem. Sci.* **2023**, *14*, 6915–6929.

(18) *Creating UV/Visible Plots from the Results of Excited States Calculations*. https://gaussian.com/uvvisplot/ (accessed 2024-03-13).
